# Global activation of oncogenic pathways underlies therapy resistance in diffuse midline glioma

**DOI:** 10.1186/s40478-020-00992-9

**Published:** 2020-07-17

**Authors:** M.-M. Georgescu, M. Z. Islam, Y. Li, M. L. Circu, J. Traylor, C. M. Notarianni, C. N. Kline, D. K. Burns

**Affiliations:** 1 NeuroMarkers PLLC, Houston, TX 77025 USA; 2 Department of Pathology, Louisiana State University Shreveport, Shreveport, LA 71103 USA; 3 Department of Microbiology and Immunology, Louisiana State University Shreveport, Shreveport, LA 71103 USA; 4 Department of Neurosurgery, Louisiana State University Shreveport, Shreveport, LA 71103 USA; 5 Department of Pediatrics, University of California San Francisco, San Francisco, CA 94158 USA; 6 Department of Pathology, The University of Texas Southwestern Medical Center, Dallas, TX 75390 USA

**Keywords:** Diffuse midline glioma, Autopsy, Next generation sequencing (NGS), Copy number variation (CNV), Proteomics

## Abstract

Diffuse midline gliomas (DMGs) are aggressive pediatric brain tumors with dismal prognosis due to therapy-resistant tumor growth and invasion. We performed the first integrated histologic/genomic/proteomic analysis of 21 foci from three pontine DMG cases with supratentorial dissemination. Histone H3.3-K27M was the driver mutation, usually at high variant allele fraction due to recurrent chromosome 1q copy number gain, in combination with germline variants in *ATM, FANCM* and *MYCN* genes. Both previously reported and novel recurrent copy number variations and somatic pathogenic mutations in chromatin remodeling, DNA damage response and PI3K/MAPK growth pathways were variably detected, either in multiple or isolated foci. Proteomic analysis showed global upregulation of histone H3, lack of H3-K27 trimethylation, and further impairment of polycomb repressive complex 2 by ASXL1 downregulation. Activation of oncogenic pathways resulted from combined upregulation of N-MYC, SOX2, p65/p50 NF-κB and STAT3 transcription factors, EGFR, FGFR2, PDGFRα/β receptor tyrosine kinases, and downregulation of PHLPP1/2, PTEN and p16/INK4A tumor suppressors. Upregulation of SMAD4, PAI-1, CD44, and c-SRC in multiple foci most likely contributed to invasiveness. This integrated comprehensive analysis revealed a complex spatiotemporal evolution in diffuse intrisic pontine glioma, recommending pontine and cerebellar biopsies for accurate populational genetic characterization, and delineated common signaling pathways and potential therapeutic targets. It also revealed an unsuspected activation of a multitude of oncogenic pathways, including cancer cell reprogramming, explaining the resistance of DMG to current therapies.

## Introduction

Diffuse midline glioma with histone H3-K27M mutation (DMG/K27M) is a newly defined entity in the World Health Organization (WHO) group of grade IV diffuse gliomas, usually seen in the pediatric population [1]. Most of these tumors occur in the pons, as diffuse intrinsic pontine gliomas (DIPG), and a smaller proportion occur in the thalamus, cerebellum or spinal cord [2]. The prognosis is dismal, with a median patient survival of 1 year, very similar to glioblastoma. In contrast to glioblastoma, even subtotal surgical resection is not possible due to the tumor location in vital brain areas. The treatment therefore relies on radiotherapy, usually in combination with chemotherapy and/or novel immunotherapies [3–6]. After initial therapy, a relatively brief period of tumor regression may be seen, after which the remaining tumor cells regrow and invade at distance from the primary focus, establishing secondary foci resistant to any therapy.

The discovery of histone H3-K27M mutations in a large subset of pediatric DMGs [7, 8] was underscored by a significantly poorer survival than in the non-mutated subset [9], although this survival difference has not been confirmed in a recent large study [10]. The p.K27M mutation occurs predominantly in *H3F3A*, encoding histone H3.3, and rarely in *HIST1H3B/C* genes, encoding H3.1 and usually affecting younger patients with better prognosis [11, 12]. The genetic landscaping has revealed mutations in other genes, such as *TP53*, *ATRX* and *ACVR1,* the last usually occurring in conjunction with H3.1 mutations in approximately 20% of DIPG cases [11]. Receptor tyrosine kinase (RTK) gene gain/amplification and pathogenic mutations were also found, especially of *PDGFRA* and *MET* [9, 13–16]. Most of these studies relied on pontine biopsies, and only limited studies addressed the spatiotemporal evolution of DIPG, mainly within infratentorial foci [13, 17].

We present here the spatiotemporal evolution of DIPG with *H3F3A* p.K27M driver mutation in three patients with complex infratentorial and supratentorial involvement. The genetic landscaping delineated known and novel recurrent chromosomal alterations and gene mutations. Importantly, all three patients harbored germline mutations in genes previously shown to increase the risk for cancer development. The proteomic analysis, a field previously unexplored in DIPG, interrogated oncogenic and cell invasion pathways. Our integrated results delineated novel migration patterns during DIPG progression and showed activation of many oncogenic pathways, deriving new considerations for candidate therapeutic targets.

## Material and methods

### Autopsy, histology and tumor burden quantification

The autopsies were performed as previously described [18], in accordance to hospital regulations. The parents consented the patients’ autopsies for diagnosis and research. The nursing team ensured prompt post-mortem transportation for autopsy. A new standardized sampling and tumor burden quantification protocol was applied. The latest MRI studies were available for all patients and used to guide autopsy sampling. The fresh brain was weighed and compared to normal standards [19]. Fresh and pilot overnight formalin-fixed paraffin-embedded (FFPE) sections were harvested from primary and secondary tumor foci, as well as from apparently normal brain and pituitary gland. The brains were subsequently fixed in 20% formalin for 10–12 days, and extensively sampled to include the following structures: whole multiple levels of the cervical spinal cord and medulla, and complete axial sections of the pons and midbrain. Bilateral samples were also harvested for paired structures: cerebellar hemispheres, thalami, basal ganglia and hippocampi. Additional samples included frontal and occipital cortex, anterior body and splenium of the corpus callosum, and fornix. Care was taken to include sections from the walls of all of the ventricles. FFPE sections were stained with hematoxylin-eosin (H&E). For patient F10, the initial biopsy was available for comparison. Images were acquired at various magnifications with Aperio Scanscope CS2 whole slide image system (Leica Biosystems, San Diego, CA) or with Nikon Eclipse Ci microscope equipped with Nikon Digital Sight DS-Fi2 camera (Nikon Instruments Inc., Melville, NY), as previously described [20, 21]. The histologic tumor burden was quantified on a 0-to-4 scale, with 0 representing no involvement, 1 – invasion by single cells, 2 – moderate cellularity, 3 – high cellularity, and 4 – massive involvement with either tumor necrosis or microvascular proliferation. Numerical data were represented graphically by using GraphPad Prism (Version 8.3.0, GraphPad Software, La Jolla, CA).

### Immunohistochemistry (IHC)

IHC was performed on selected sections, as described [20, 22]. Primary antibodies were: histone H3-K27M (Millipore/Sigma, Burlington, MA), IDH1-R132H (DIA-H09, Dianova, Hamburg, Germany), p53 (DO-7), vimentin (V9), Ki-67 (30–9) (Roche/Ventana, Tucson, AZ), Olig-2 (387 M-15), GFAP (EP672Y) (Ventana/Cell Marque, Rocklin, CA).

### Transmission electron microscopy

Freshly collected autopsy samples were processed as previously described [20, 23].

### Next generation sequencing (NGS)

Nucleic acids were extracted from fresh frozen or FFPE samples, as described [22]. FFPE microdissection was performed on selected samples either to separate morphologically different neoplastic populations or tumor cells from normal cells. Matched normal tissue was obtained either from pituitary gland (F5 and F10), or uninvolved frontal cerebral cortex (F12). All samples were sequenced by using a customized 295-gene DNA library (SureSelect XT-HS, Agilent, Santa Clara, CA), as described [22, 23]. For all F5 and F12 samples, and for F10 ganglion-like cells (GCs), NGS was also performed at Tempus Labs (Chicago, IL), by using the xT 596-gene panel, as described [22, 24]. The two F10 biopsies were additionally sequenced at University of California/San Francisco. Variant analysis and interpretation were performed as described [22–24]. Copy number variation (CNV) analysis was performed at Tempus Labs [25]. Loss of heterozygosity (LOH) refers to alterations with loss of one allele. Tumor mutation burden (TMB) represents the number of single nucleotide protein-altering mutations per million base pairs.

### Proteomic analysis

Fresh frozen tissue lysis and Western blotting (WB) were performed as described [26–28]. Briefly, 25–50 mg frozen tissue was homogenized on ice with 500 μl Tissue Protein Extraction Reagent (T-PER) reagent (Pierce Biotechnology, Rockford, IL) containing Halt Protease Inhibitor Single-use Cocktail, EDTA-Free (Pierce Biotechnology). Following centrifugation at 10,000×*g* for 5 min, the supernatant was collected, protein concentration was measured and the samples were denatured and resolved by SDS-polyacrylamide gel electrophoresis. Multiple primary antibodies were tested for the same protein, and the ones showing the highest specificity were selected for WB analysis (Suppl. Table S1). Positive controls for specificity testing included previously tested glioblastoma cell lines [28], and patient samples with known mutations. The WBs for each antibody were repeated at least twice, with similar results. The densitometric analysis was performed by scanning the X-ray films with optimal exposures on a ChemiDoc™ Touch imager (Bio-Rad, Hercules, CA). The bands were further quantified by using the Image Lab 6.0 software (Bio-Rad). Individual protein values were normalized to the corresponding actin values, except for phosphoprotein values that were normalized to the corresponding unphosphorylated protein values. Minus values were manually adjusted as zero. Results were expressed as fold-increase or fold-decrease compared to normal control.

## Results

### Clinical overview of three DIPG patients

In order to understand disease progression and resistance to therapy in DMG/K27M, we performed an integrative comparative analysis of three autopsies of 5-, 10- and 12-year-old White females (F5, F10, F12) with normal psychomotor development, normal weight, no co-morbidities, and residing within the same geographic area (Table 1).

**Table 1:** Patient clinical data

Sex/age^a^	Symptoms at presentation	Survival months	Bx	Rx	Chemotherapy & vaccine	Primary focus^b^	Secondary foci^b^ (months)
**F5 (3)**	Headache	20	No	2x	PARP1/2 inh	Pons	R cerebellum (14)
	Anger				Carboplatin&		
	CN 6				HDAC inh (CED)		
**F10 (9)**	Headache	13.5	Yes	2x	K27M vaccine	Pons	R&L cerebellum
	CN 7, 8				Nivolumab		R&L LVs (9)
					HDAC/PIK3CA inh		
**F12 (11)**	Headache	13	Yes	1x	Erlotinib	Pons	R cerebellum
	CN 7, 8				Bevacizumab		R LV

*F* Female, *CN* Cranial nerve, *Bx* Biopsy, *Rx* Radiotherapy, *HDAC* Histone deacetylase, *inh* Inhibitor, *CED* Convection enhanced delivery, *R* Right, *L* Left, *LV* Lateral ventricle

^a^The age is given in years: at death/autopsy following the sex; at initial presentation in parenthesis

^b^The primary and secondary foci, as visualized by MRI. The progression time to secondary foci is shown in months, when recorded

F5 initially complained of daily headaches and recurrent ear and sinus infections, followed by behavioral changes manifested by extreme anger, and right eye strabismus. Magnetic resonance imaging (MRI) showed a 5.5 × 5.0 × 3.9 cm, non-enhancing, pontine mass with secondary hydrocephalus of 4th and 3rd ventricles that was radiologically diagnosed as DIPG without biopsy. She initially received 6 weeks of radiotherapy and veliparib (PARP1/2 inhibitor), followed over several months by 8 carboplatin and HDAC inhibitor infusions by convection enhanced delivery with significant tumor regression. Fourteen months post-diagnosis, the patient presented with dysphagia, and MRI showed tumor regrowth and extension to right cerebellum (Suppl. Fig. S1A). F5 expired 20 months post-diagnosis and the definitive diagnosis of DMG/K27M, WHO grade IV, was rendered at autopsy.

F10 presented with chronic headaches, hearing loss and subsequent left facial droop. MRI showed a 5.3 × 4.4 × 3.9 cm, rim-enhancing, pontocerebellar mass, with 4th ventricle compression, tonsillar herniation and 3rd and lateral ventricles dilatation (Suppl. Fig. S1B). A biopsy rendered the diagnosis DMG/K27M, WHO grade IV. The patient received 6 weeks of initial brainstem radiotherapy, followed by K27M-peptide vaccine therapy (7 cycles; 3 weeks/month) until week 24, when MRI showed progression by involvement of supratentorial periventricular brain structures (Suppl. Fig. S1C). A repeat frontal corpus callosum biopsy was performed at 10 months post-diagnosis, confirming secondary tumor involvement. F10 further received frontal area proton beam therapy (3 cycles) and nivolumab (3 cycles). At this time, she presented chronic left 6th and 7th cranial nerve palsy, and abnormalities of coordination and gait. She further received CUDC907, a dual HDAC and PI3K-p110α inhibitor (1 cycle), but her status declined abruptly and she expired 13.5 months post-diagnosis.

F12 presented with headache, left-sided facial weakness and balance disturbance. MRI showed a large pontine mass that was biopsied and diagnosed as glioblastoma, WHO grade IV. She received 6 weeks of radiotherapy and concurrent erlotinib and bevacizumab chemotherapy. An MRI performed 11.5 months post-diagnosis showed tumor progression with bilateral cerebellum and right frontal periventricular involvement (Suppl. Fig. S1D). She expired 13 months post-diagnosis, and was retrospectively diagnosed at autopsy with DMG/K27M, WHO grade IV.

### Patterns of histologic invasion in DIPG

**Fig. 1:**
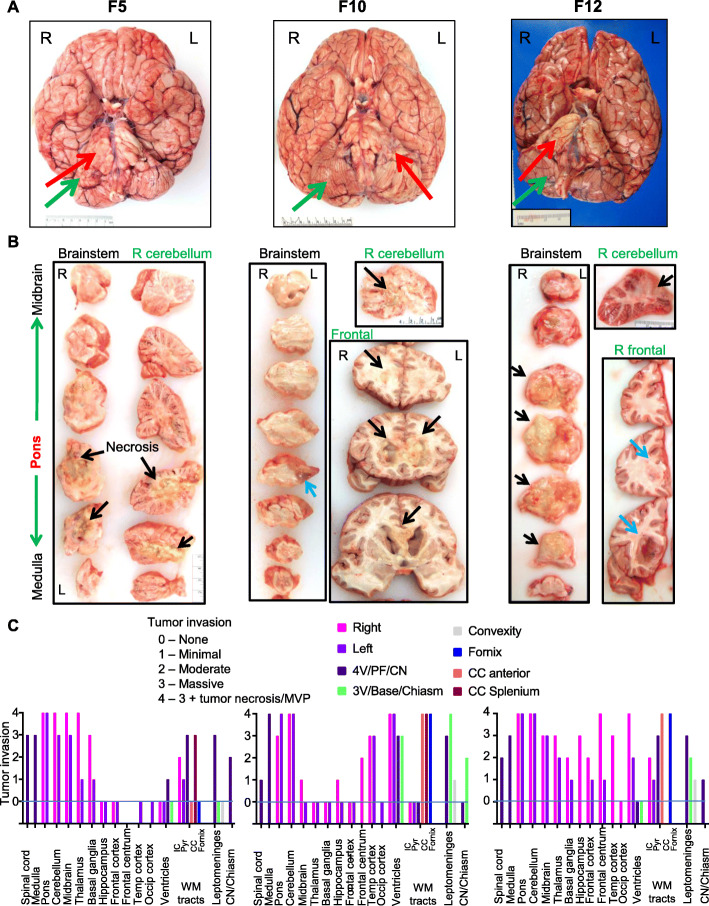
Histologic patterns of tumor cell invasion in DIPG. **a.** Brain gross appearance. Red and green arrows indicate the primary pontine and the secondary cerebellar foci, respectively. R, right; L, left. **b.** Sections of indicated brain structures with gross visible tumor foci. Black and blue arrows indicate liquefying necrosis and fleshy tumor, respectively. **c.** Semiquantitative analysis of histological tumor involvement of the specified color-coded structures. MVP, microvascular proliferation; 4 V, 4th ventricle; PF, posterior fossa; CN, cranial nerve; 3 V, 3rd ventricle; CC, corpus callosum; Temp, temporal; Occip, occipital; WM, white matter; Pyr, pyramidal tracts; IC, internal capsule. The panels and graphs from (**b**) and (**c**), respectively, correspond to the brains from (**a**)

Brain gross examination showed diffuse enlargement of the pons in all cases, and regional foliar expansion and effacement in the right cerebellar hemisphere (Fig. 1a). The F10 fresh brain had increased weight (Suppl. Table S2), with mild gyri flattening, consistent with cerebral edema. All cases presented with shunted hydrocephalus and F5 had right cerebellar tonsil herniation (Suppl. Table S2). Brain sectioning showed liquefying necrosis in all tumor foci noted radiologically (Fig. 1b, black arrows), except for F10 pons and F12 right frontal focus that appeared fleshy, red, and indurated (Fig. 1b, blue arrows).

Tumor burden digital quantification (Fig. 1c; Suppl. Fig. S2) delineated distinct cell dispersal patterns: (1) centrifugal parenchymal infiltration from the primary pontine focus in F5, (2) massive seeding of ventricles and leptomeninges with minimal neuropil infiltration in F10, and a mixed pattern in F12 that showed the highest tumor burden.

**Fig. 2:**
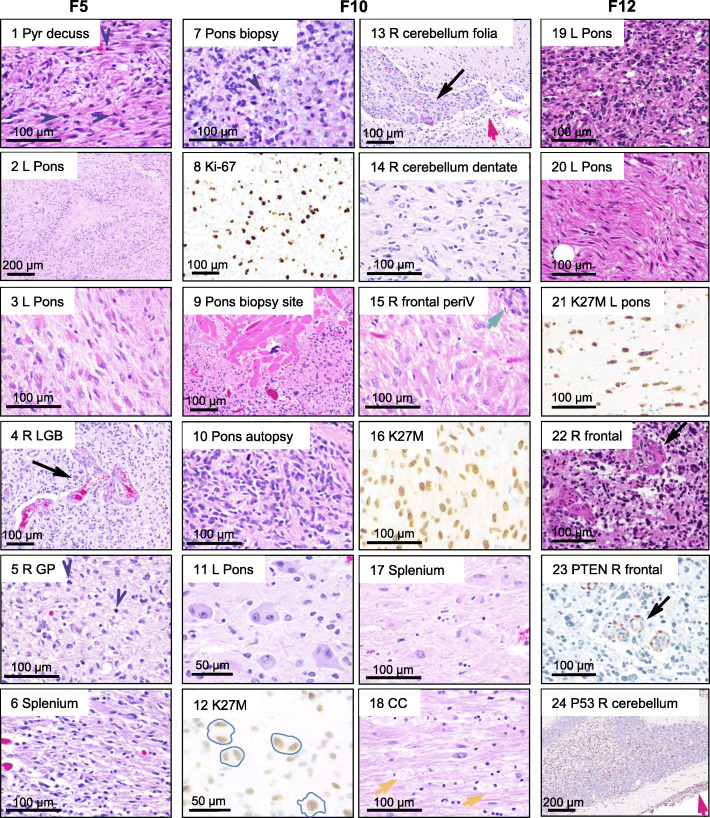
Morphologic spectrum of the 3 DIPGs across infratentorial and supratentorial foci. H&E and IHC with Ki-67, histone H3-K7M, PTEN and p53 antibodies of selected sections indicated by numbers and anatomic locations. The columns correspond to each case, with two central columns shown for F10. L, left; R, right; Pyr decuss, pyramidal tract decussation; LGB, lateral geniculate body; GP, globus pallidus; periV, periventricular; CC, corpus callosum. Blue arrowheads and black, red, green and yellow arrows indicate mitotic figures, microvascular proliferation, leptomeningeal spread, subventricular ependymal cell proliferation, and foamy macrophages, respectively. Among other features, note various neoplastic cell morphologies, supratentorial involvement for all tumors, brisk mitotic activity in multiple F5 foci and in the F10 biopsy, viable striated muscle fibers in F10, most likely at prior biopsy site (panel 9), labeling with H3-K27M antibody of F10 bi-nucleated ganglion cells (blue contour in panel 12), and PTEN expression detected in endothelial cells but not in tumor cells, in F12

At least two morphologically distinct populations were apparent in each case (Fig. 2; Suppl. Fig. S3). F5 and F10 showed areas of small neoplastic cells, with elongated or irregular nuclei and scant cytoplasm (Fig. 2, F5: panels 1–2, 4–6; F10: panels 10, 13–14), and areas of larger neoplastic cells, with abundant eosinophilic cytoplasm, resembling reactive astrocytes (Fig. 2, F5: panel 3; F10: panels 15, 17–18, Suppl. Fig. S3B). The initial F10 pontine biopsy showed similar morphology to the postmortem pontine sections except for the presence of a myxoid background (Fig. 2, compare panels 7 to 10). A third unexpected morphology of large, dysplastic ganglion-like cells (GCs), as in ganglioglioma, previously reported in DMG/K27M [29], with H3-K27M expression but lacking *BRAF* mutations, was present in F10 pons focally (Fig. 2, panels 11–12, Suppl. Fig. S4), demonstrating the metaplastic potential of these neoplastic cells. An F10 incidental autopsy finding was the presence of striated muscle fibers surviving within the tumor at the site of the previous biopsy, most likely carried by the surgical procedure (Fig. 2, panel 9). F12 showed predominantly glioblastoma-like pleomorphic cells (Fig. 2, panels 19, 22, Suppl. Fig. S3C), and focally in pons, elongated bipolar neoplastic cells (Fig. 2, panel 20, Suppl. Fig. S3D). Common features for all tumors were pseudopalisading necrosis and microvascular proliferation. Leptomeningeal invasion was developed in F10 and F12 (Fig. 2, panels 13&24, red arrows). Mitotic activity was brisk in F5 sections (Fig. 2, panels 1, 5, arrowheads), but scant in F10 and F12 postmortem sections.

All neoplastic cells expressed H3-K27M mutant by IHC (Fig. 2; Suppl. Fig. S5, Suppl. Table S3). IDH1-R132H was negative in all cases, p53 expression was high in F12 (Fig. 2, panel 24), but not in F5 or F10 (Suppl. Fig. S5), and PTEN expression loss was noted in F12 neoplastic cells (Fig. 2, panel 23). Olig-2 was expressed in F5 but not in F10 (Suppl. Table S3). GFAP labeled F5 and F10, but not F12 neoplastic cells, whereas vimentin was mostly negative in F5 and F12 neoplastic cells, and positive in the F10 small neoplastic cells (Suppl. Fig. S5).

### NGS revealed germline and progressive somatic alterations in DIPG

**Fig. 3:**
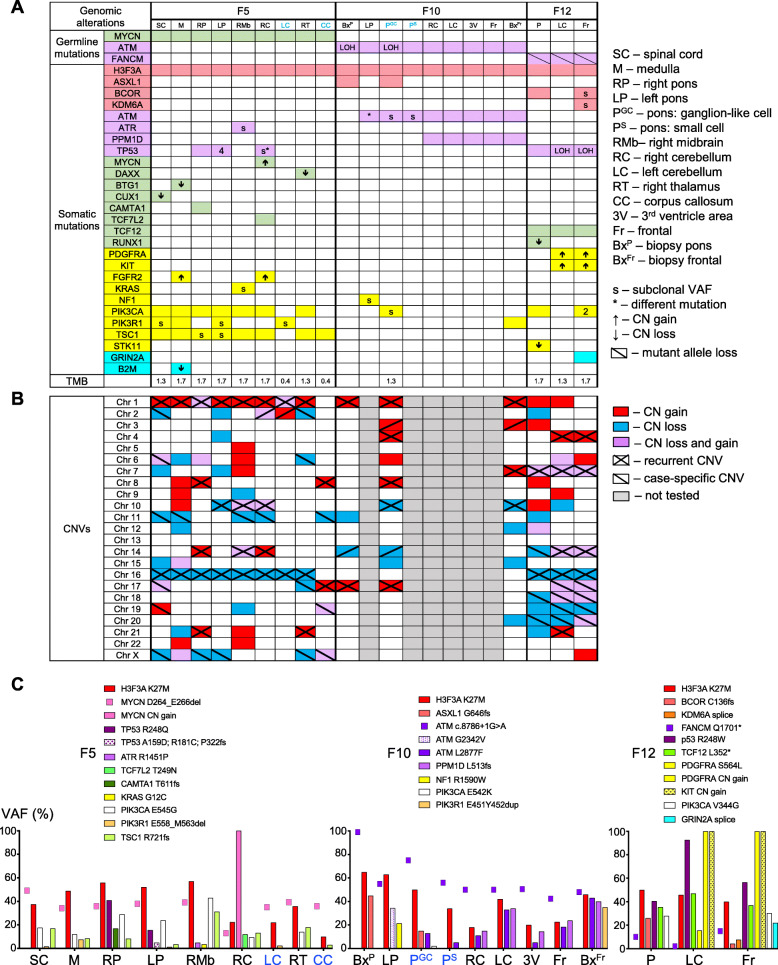
Spatio-temporal genomic profiling. **a.** Pathway color-coded representation of gene mutations and selected CNVs: chromatin remodeling – pink; DDR – purple; transcription factors and modulators – green; growth factor receptors and mediators – yellow; other – blue. White boxes lack mutations, and numbers in boxes indicate the number of mutant variants for the respective gene. FFPE microdissected tumor foci are showed in blue font and show decreased TBM for the F5 samples, indicating limited tumor material available for NGS that most likely lowered the mutation and CNV detection rate in these samples. **b.** Color-coded CNV analysis. **c.** VAF graphic representation: germline mutations as dots, somatic mutations as bars. FFPE-microdissected samples are shown in blue font and show decreased VAF for the F5 samples

NGS was performed in the primary pontine and secondary infratentorial and supratentorial foci, two F10 biopsies, matching normal tissue, totaling 21 tumor and 3 normal samples. All the patients had confirmed or potentially deleterious germline mutations in cancer-associated genes (Fig. 3; Suppl. Table S4). F5 harbored a heterozygous germline *MYCN* in-frame deletion of three residues p.264_DDE_266, mapping to the conserved central acidic protein region that is a CKII phosphorylation target [30]. Hypothetically, this deletion may generate a consensus p53-binding motif that may lead to p53 inactivation similarly to viral oncogenes. Interestingly, *MYCN* normal allele CN gain occurred in the right cerebellar focus (Fig. 3; Suppl. Table S5). F10 presented heterozygous germline *ATM* splice-site mutation predicted to truncate the carboxyl (C)-terminus and classified as germline pathogenic mutation for ataxia-telangiectasia (AT) syndrome (ClinVar, multiple submitters). This germline mutation was accompanied by normal allele somatic inactivation through various mechanisms (see below), consistent with a tumor suppressor role for *ATM*. F12 harbored a heterozygous germline *FANCM* nonsense mutation, p.Q1701*, predicted to truncate FANCM C-terminus, and shown to confer increased risk for breast cancer development [31]. Paradoxically, this mutant allele was lost in all the tumor foci, by chromosome 14q CN loss (Fig. 3; Suppl. Table S5).

*H3F3A* p.K27M was the only somatic aberration present in all tumor foci from all cases (Fig. 3a). Its variant allele fraction (VAF) was higher than that of other heterozygous gene alterations in F5 and F10, most likely due to CN gain of the mutant *H3F3A* allele by a recurrent 1q gain (Fig. 3b-c; Suppl. Table S5). Somatic mutations in three other chromatin remodeling genes, *ASXL1, BCOR* and *KDM6A*, were detected in isolated foci from F10 and F12 (Fig. 3a). Although *ASXL1* mutations are usually seen in non-glial neoplasms [32], rare mutations have been described in DMG/K27M [33]. Interestingly, the pathogenic protein-truncating *ASXL1* mutation was solely detected in the F10 pontine biopsy and pontine autopsy GC population (Fig. 3a), suggesting common derivation. BCOR, is a component of the noncanonical polycomb repressive complex (PRC) 1.1 [34], and *BCOR* mutations have been described focally in DIPG with H3.3-K27M mutation [13]. In F12, a *BCOR* frameshift loss of function mutation was detected in the pontine focus and also in the frontal focus at subclonal VAF. KDM6A is a di- and trimethyl H3-K27 demethylase that modulates the recruitment of PRC1 and the monoubiquitination of histone H2A [35]; *KDM6A* alterations have not been previously described in DIPG. An inactivating *KDM6A* splice variant was detected at subclonal VAF in F12 frontal focus (Fig. 3; Suppl. Table S4).

Somatic pathogenic mutations in the DNA damage response (DDR) pathway were present in all three cases (Fig. 3; Suppl. Table S4). In F12, the *TP53* p.R248W pathogenic mutation with or without LOH was detected in all tumor foci. In F5, as many as five clonal or subclonal *TP53* mutations were detected in pontine and right cerebellar foci. In F10, a gain-of function truncating mutation in exon 6 of *PPM1D* that encodes the wild-type p53-induced phosphatase 1D (WIP1) was detected at clonal VAF in the cerebellar and supratentorial foci. PPM1D/WIP1 is a phosphatase that dephosphorylates and inactivates many DDR mediators, such as ATM, p53, CHK2 and H2AX, and truncating exon 6 mutations were previously reported in DIPG as mutually exclusive with *TP53* mutations [36–38]. In addition, F10 harbored second-hit inactivation of the normal *ATM* allele by different mechanisms in different foci: (1) LOH by chromosome 11q loss containing the *ATM* locus, for the pontine biopsy and autopsy GC populations, (2) p.L2877F somatic mutation at clonal VAF, for the cerebellar and supratentorial foci, and (3) p.G2342S somatic mutation, for the left pontine population. The L2877F somatic mutation was previously reported as pathogenic mainly in lung adenocarcinomas [39], and a germline G2342S variant of uncertain significance (VUS) was reported in the AT syndrome (ClinVar/Invitae).

Somatic mutations in transcription factors or modulators involved in cell differentiation and cell cycle were detected in F5 and F12 (Fig. 3a). Most of the alterations were focal, due to homozygous CN loss in *DAXX*, *BTG1*, *CUX1*, *RUNX1* (Suppl. Table S5), *MYCN* amplification, *CAMTA1* frameshift or *TCF7L2* missense mutation (Suppl. Table S4). A somatic *TCF12* p.L352* nonsense mutation present in all F12 tumor foci at heterozygous VAF suggested early co-occurrence with the *H3F3A* p.K27M mutation, as well as selective advantage (Fig. 3c). This mutation truncates half of the protein, including the C-terminal DNA-binding bHLH domain that is required for transcriptional activity [40]. Although *TCF12* mutations were not previously reported in DIPG, they were shown in anaplastic oligodendroglioma to correlate with more aggressive tumor types [40].

Somatic alterations of the RTK/PI3K/MAPK/mTOR pathways were also seen in all cases, although very focal in F10 (Fig. 3; Suppl. Tables S4-S5). *PDGFRA* and *KIT* amplification was detected in F12 cerebellar and frontal foci. An oncogenic *PDGFRA* p.S564L missense mutation mapping to the juxtamembrane region, previously described in colorectal carcinoma [41], was present at low VAF in F12 cerebellar tumor. *PIK3CA* p.E545G and p.V344G pathogenic mutations mapping to hot spots in the C2 and helical domains of PI3K-p110α catalytic subunit, respectively, were detected in almost all F5 and F12 foci, respectively. A *PIK3CA* p.E542K mutation was detected at very low VAF in the F10 GC population. Pathogenic *PIK3R1* p.E558_M563 in frame deletion and p.E451_Y452 in-frame duplication mapping to the inter-SH2 region of PI3K-p85 regulatory subunit were detected in a left-sided F5 infratentorial subpopulation and in F10 frontal biopsy, respectively. Although not identical, similar oncogenic mutations have been reported in endometrial cancer [39, 42]. A *KRAS* gain-of-function mutation was detected focally in an F5 midbrain subpopulation. An *NF1* R1590W somatic missense mutation mapping to the Sec14-PH domain and previously described in syndromic NF1 [43] was detected focally in F10 pons. A *TSC1* pathogenic frameshift mutation was detected throughout F5, suggestive of early occurrence and selective advantage. An *STK11* homozygous CN loss was noted in F12 pons.

Focal somatic pathogenic alterations occurred in *B2M* and *GRIN2A* genes encoding transmembrane proteins (Fig. 3a), the latter gene being mutated in metastatic glioblastoma [24] and frequently, in melanoma [44]. Other VUS were detected in F12: *EGFR* p.A613T at low VAF in frontal focus, and *STAT4* p.E194* at heterozygous VAF in pontine and frontal foci (Suppl. Table S4).

The TMB in various tumor foci from all three cases showed little variation, with values between 1.3 and 1.7 mutations/MB (Fig. 3a), ranking at the lower end for various cancer types, as previously described [45].

The CNV analysis showed a combination of CN gains and losses in all cases, consisting of recurrent, case-specific and isolated CNVs (Fig. 3b; color-coded Suppl. Table S5). Recurrent CNVs, called if detected in at least two cases, were either (1) gains of 1p-distal-end; 1q, including the *H3F3A* locus; 4q12, mapping to *PDGFRA* and *KIT* loci; 7q, containing *SERPINE1* that encodes plasminogen activator inhibitor 1 (PAI-1); 8q-distal-end; focal 14q11.2, encompassing *TRA* (T cell receptor alpha) locus; 17p13, including *CRK* proto-oncogene involved in glioblastoma invasiveness [46, 47]; 17q25; 21q11-q21, or (2) losses of 10q-distal-end, encompassing *ECHS1* encoding enoyl-CoA hydratase-1, whose deficiency has been shown to activate mTOR [48]; 16q, including *PHLPP2* tumor suppressor. Entire chromosome 14 loss was detected in F10 pontine biopsy and GC samples, and 14q loss, in all F12 samples. Focal 10q25-q26 CN gain was detected in three F5 samples and the pontine F12 sample, and contained high or low gain depending on *FGFR2* presence or absence, respectively. Many of these recurrent CNVs are novel, compared to a previous report describing CNVs in DIPG [49]. Case-specific CNVs were noted in some or all samples from a given case, e.g. F5 and F12 contained as many as 7 or 6 different case-specific CNVs, respectively, each detected in at least two samples. F12 showed the most extensive and complex case-specific CNVs, including complex alterations on chromosome 7 in all samples, with additional LOH in the cerebellar and frontal foci, and loss of chromosome 18 in all samples, with focal high CN gain of distal 18p, containing *TYMS* (thymidylate synthetase), in the cerebellar and frontal foci. The 17p13 chromosomal region containing *CRK* and *TP53*, showed low CN gain in F5 and F10, sometimes without involving *TP53*, and an interesting evolution pattern in F12: neutral LOH in pons, *CRK* neutral LOH and *TP53* heterozygous CN loss in cerebellum, and entire region heterozygous CN loss in the frontal focus, suggesting a role for *CRK* in DIPG invasion. Except for the F5 midbrain sample, all individual samples contained isolated CNVs; e.g. chromosome 10 loss, a recurrent CNV in glioblastoma, was present only in F12 cerebellar sample.

### Activation of epigenetic, DDR, cell growth and migration pathways in DIPG

**Fig. 4:**
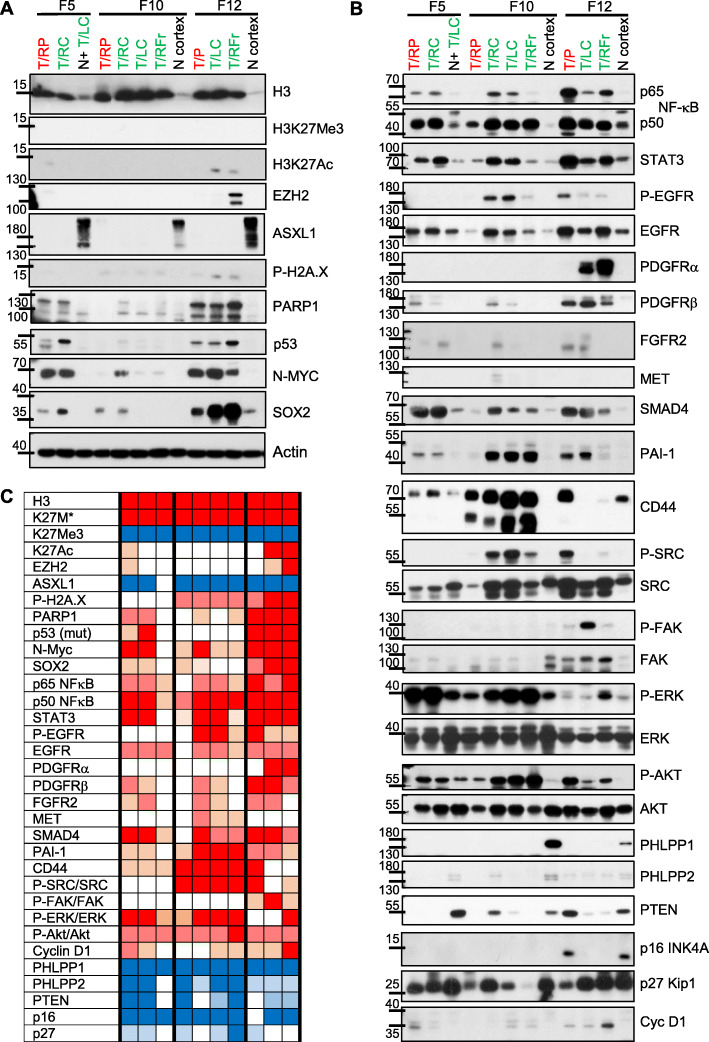
Proteomic profiling of DIPG foci. **a-b.** WB analysis with indicated antibodies of total protein lysates (50 μg proteins) from tumor (T) and normal (N) fresh frozen autopsy samples. Primary pontine (P) foci are indicated in red font and secondary cerebellar (C) and frontal (Fr) foci, in green font. Sample laterality: R, right; L, left. Supplemental Fig. S6 shows the positive control for H3 K27Me3 antibody from an adult glioblastoma autopsy case with EZH2 overexpression. Note lack of H3 K27 methylation in the presence of EZH2 overexpression for F12 right frontal (RFr) tumor focus. **c.** Heat map of semiquantitative WB analysis, as shown quantified and normalized to loading controls in Supplemental Fig. S6. Shades of red and blue indicate expression level increase or decrease, respectively, as compared to normal controls

To profile pathway activation in DIPG, a semiquantitative proteomic analysis was undertaken in grossly apparent tumor foci and control normal cortex (Fig. 4; Suppl. Fig. S6). Global epigenetic changes included strong histone H3 upregulation, absent H3-K27 trimethylation, undetectable EZH2 expression, except for the F12 frontal focus that showed overexpression, and ASXL1 downregulated levels, without mutations in these samples. Focal H3-K27 acetylation was detected in F5 and F12, but not F10.

DDR involves an early step of histone variant H2A.X phosphorylation by ATM [50]; phospho-Ser139 H2A.X was minimal in F5, but was relatively high in F12 foci. Surprisingly, it was also detected with lower intensity in all F10 tumor foci with bi-allelic *ATM* inactivation, suggesting compensation by a related kinase. PARP1 levels were increased in F5 foci (F5 received an initial course with PARP1/2 inhibitors), strongly increased in all F12 foci, and minimally increased in some F10 foci. Elevated p53 levels were present in F5 and F12 tumor foci that harbored *TP53* missense mutations known to stabilize the mutant protein [51]. N-MYC transcription factor has been implicated in pathogenesis of high-grade pediatric gliomas, including DIPG without H3-K27M mutation [11]. It showed high levels in F5 right pons and cerebellum, correlating with the detected *MYCN* amplification in the latter focus, but not in the normal sample, suggesting that the germline *MYCN* alteration does not influence protein expression. N-MYC was also strongly upregulated in all F12 foci and in F10 right cerebellar tumor that did not harbor gene amplification, suggesting increased transcription, protein stability, or both. SOX2, a transcription factor essential for maintaining undifferentiated neural stem cells [52], was upregulated in all three DIPGs, although patchy in F10, and with high overexpression in F12 cerebellar and frontal foci.

The levels of many oncoproteins were upregulated in DIPGs, either in generalized or more focal patterns. The heterodimeric NF-κB transcription factor proteins p50 and p65 were upregulated in all three cases in many foci. Similarly, STAT3 transcription factor was globally upregulated. Among the RTKs tested, EGFR showed upregulation in all cases, but with mild to moderate phosphorylation only in F10 and F12 foci. PDGFRα was strongly upregulated in F12 cerebellar and frontal foci, reflecting the *PDGFRA* amplification detected in these samples (see Fig. 3), and PDGFRβ was mildly or moderately upregulated in multiple foci. FGFR2 was mildly to moderately upregulated in foci from all three cases and correlated with CN gain in F5 right cerebellar focus (Suppl. Table S5). MET was mildly upregulated in F10 cerebellar foci.

Known mediators of glioma invasion pathways showed also increased expression levels. SMAD4, the common transducer of TGF-β and bone morphogenic protein pathways, and its downstream target PAI-1 [53], both involved in glioma invasion and angiogenesis [54, 55], were upregulated in nearly all foci. In contrast to PAI-1 upregulation that may be partly due to recurrent 7q CN gain, *SMAD4* showed CN loss in all F12 foci due to chromosome 18 loss (Suppl. Table S5). Its increase is most likely controlled by epigenetic transcriptional regulation and protein turnover mechanisms. CD44, a hyaluronic acid receptor that associates and promotes c-SRC activation [56] and is involved in glioma cell invasion [57, 58], was upregulated in almost all foci and correlated with c-SRC activation. In contrast, FAK showed focal strong activation in the F12 cerebellar focus.

As expected from the overall upregulation of RTKs, the ERK/MAPK and PI3K/AKT pathways were activated in virtually all foci. The phosphorylated ERK1–2 species in F12 pontine and cerebellar foci are not shown or quantified, but were present as strong bands shifted upward, most likely due to tissue degradation, and, as expected, did not react with other MAPK family members, including JNK (not shown). The expression levels of selected tumor suppressors showed global or more focal decrease. PHLPP1/2 proteins that are PI3K/AKT [27, 59] and NF-κB pathway inhibitors [26], were globally downregulated, and their respective genes showed monoallelic loss by case-specific or recurrent chromosome 18 and 16q loss, respectively (Suppl. Table S5). PTEN tumor suppressor was downregulated in most F5, F10 and F12 foci. However, contamination by normal neurons containing high PTEN expression levels is hard to avoid and cannot be excluded in cerebellar and pontine samples. The two cyclin D-CDK4/6 complex cell cycle inhibitors showed different expression trends: p16 INK4A, encoded by *CDKN2A,* was downregulated in all foci, except for the F12 pontine focus that showed also PTEN retention, whereas p27 Kip1 was not significantly decreased except for the F10 frontal focus. Conversely, cyclin D1 expression levels were mildly increased in almost all foci.

## Discussion

DMG/K27M are therapy-resistant aggressive tumors with extremely poor survival, occurring mainly in the pediatric population [2]. Tremendous therapeutic efforts to achieve tumor growth control [3–6] are coupled to clinicopathologic efforts for prognostic subgrouping [9–12]. A better understanding of the natural evolution of the disease, especially of the unrelenting dispersal of the tumor cells, has also been attempted [13, 17], but is hampered by the lack of pathological material from autopsy. To tackle this drawback, we performed the first integrated histopathologic/genomic/proteomic analysis of a large number of matched infratentorial and supratentorial tumor foci from 3 DIPG autopsies.

### Patterns of invasion in DIPG

**Fig. 5:**
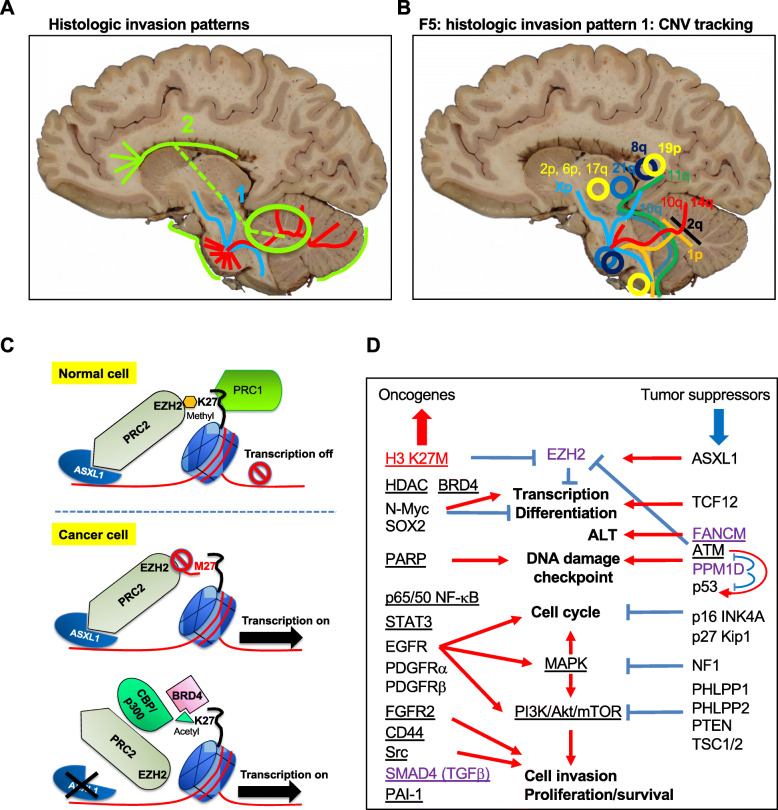
Models of invasion, pathway activation and therapeutic targeting in DIPG. **a**. Histologic invasion pathways include the common ponto-cerebellar dissemination pathway, shown in red, and the more specific centrifugal and CSF/leptomeningeal pathways shown in blue and green, respectively. The green circle indicates the cerebellar tumor as the most common origin of supratentorial CSF seeding. **b.** Spatial color-coded CNV-tracking of invasive neoplastic populations within the F5 tumor that showed histologic centrifugal migration pattern. Contiguous neoplastic populations are indicated with line tracings, and isolated foci sharing the same CNV composition, with circles. Cerebellar bars indicate bilateral spread of the respective population. The corresponding color-coded CNVs are indicated in bold for gains, and regular font, for losses. **c.** Schematic model of epigenetic changes targeting histone H3 K27 residue. The histone H2A/H2B/H3/H4 nucleosome is represented as a blue barrel, the DNA, as a red thread, and the N-terminal histone H3 “tail”, as a black curved line. The methyl and acetyl groups are shown in orange and green, respectively. The stop sign shows blockage of enzymatic or transcriptional activity. **d.** Pathway activation and drug targeting model in DIPG. The oncogenic H3 K27M driver protein and mediators with dual tumor suppressor or oncogenic roles depending on context are shown in red and purple, respectively. Otherwise, the oncogenic and the tumor suppressor proteins are shown on left and right, respectively. The pathways are shown in bold font, and red and blue indicate activation or suppression, respectively. The candidate targets for therapy are underlined

The semiquantitative histologic analysis of tumor spread suggested the presence of a common ponto-cerebellar migration pattern (Fig. 5a, red), and two secondary patterns: centrifugal parenchymal dispersal (Fig. 5a, blue-pattern-1), and CSF spread with anterograde seeding of supratentorial foci (Fig. 5a, green-pattern-2). The latter pathways may present relatively separated or may coexist, and it appears that the CSF spread correlated with shorter survival. The genomic/proteomic analysis of infratentorial and supratentorial foci confirmed and expanded these histologic invasion patterns. We have previously shown that the tumor mutation signature shows little spatiotemporal variability, being reliable for tracking the origin of invasive populations, whereas the CNV composition shows high variability in distinct tumor foci [24]. A common mutation set characterized all tumor foci in each DIPG case, and additional genetic alterations allowed tracking the spatiotemporal selection and migration of neoplastic populations. The CNVs reflected more precisely the marked heterogeneity of the various subsets of neoplastic cells and their patterns of invasion (Fig. 5b). For F5 that exhibited histologic centrifugal invasion, the pontine population showed a *TSC1* pathogenic mutation at subclonal VAF, and this mutant subpopulation was selected and subsequently migrated to all other tumor areas. However, it appears that many different recurrent or case specific CNVs occurred in this subpopulation, tracking distinct migratory subpopulations either towards the cerebellum and midbrain or cervical spinal cord (Fig. 5b, red and orange lines), or in centrifugal paths towards the spinal cord and supratentorial foci (Fig. 5b, light blue lines). Interestingly, the spinal cord and supratentorial foci shared four additional CNVs that were not detected in the brainstem or cerebellar foci (Fig. 5b, yellow circles), and the pons and supratentorial foci shared another two similar CNVs (Fig. 5b, blue circles). We speculate that these CNVs may confer a selective migration advantage to these cell populations, and listed putative invasion-promoting genes in these chromosomal regions for further confirmatory studies (Suppl. Table S5). To add to the complexity, a pattern of centrifugal migration may originate from the cerebellum and circumvent the pons, perhaps occurring later in the evolution of the tumor (Fig. 5b, green lines). For the second histologic invasion pattern, with extensive leptomeningeal and ventricular dissemination, the genetic analysis revealed many alterations common only to the cerebellar and supratentorial foci, indicating that the supratentorial seeding originates from a cerebellar rather than pontine neoplastic population (Fig. 5a, green circle). This finding challenges the conclusion from a previous study based on two autopsy cases with supratentorial foci without histological characterization stating that DIPG is a homogenous tumor, and therefore, the pontine population present in the initial biopsy is representative for the entire tumor [17]. Especially for tumors with the second invasion pattern and/or cerebellar involvement at presentation, our data showed significant spatial genetic divergence between the pontine and cerebellar or supratentorial populations. Moreover, for F10, for whom tissue was available from 2 biopsies and autopsy, neither the initial pontine biopsy nor the pontine autopsy populations were representative for the major neoplastic population that disseminated throughout the brain.

### Oncogenic pathway profiling and candidate therapies in DIPG

Histone H3.3-K27M mutation was ubiquitous, consistent with its role as driver oncogenic mutation, as previously reported [17, 60]. Moreover, the mutant allele had CN gain, an observation recently reported by others [61]. A therapeutic vaccine approach against the mutant peptide [4] has been used for F10, but did not appear to extend survival. H3-K27 residue is target to multiple posttranslational modifications, being methylated by the PRC2 catalytic components EZH1/2, and acetylated by the CBP/p300 acetyltransferase complex [62] (Fig. 5c). PRC2 is recruited in the presence of ASXL1 to unmethylated CpG islands to maintain silent genes in repressed state [63, 64]. Although an isolated *ASXL1* mutation was detected in F10, the proteomic analysis showed global ASXL1 expression downregulation, suggesting an additional common mechanism contributing to PRC2 inactivation in DIPG. H3-K27M mutant inhibits EZH2 catalytic activity in a dominant-negative manner [65], resulting in global H3-K27 hypomethylation and transcription de-repression [66–68]. The unmethylated K27 may be acetylated to further activate transcription, and we found its acetylation in F5 and F12, unrelated to prior anti-HDAC therapy. Additional chromatin remodeling genes were altered more focally, either by mutation or by CNV. *TYMS* acquired high CN gain in F12 cerebellar and frontal foci and may represent a 5-fluorouracil/capecitabine therapy target [69, 70].

DDR pathway mutations, either germline or somatic, were present either in the common genetic signature, in F10 and F12, or more focally, in F5 (Fig. 5d). *TP53*, *PPM1D* and *ATM* somatic mutations have been previously described in DIPG [11, 33, 38]. The detection of *ATM* germline mutation in F10 adds DMG/K27M to the list of malignancies for which AT syndrome carriers are at risk. The multiple mechanisms for second *ATM* allele somatic inactivation suggested independent spatial evolution from an initial pauci-mutated ponto-cerebellar population. The acquisition of a mutation in a second DDR modifier, *PPM1D*, conferred most likely a selective advantage to the population in the cerebellum that further populated the supratentorial compartment. PPM1D/WIP1 activation is expected to result in further ATM and p53 inactivation [71] with suppression of DDR and cell cycle check points (Fig. 5d). Surprisingly, effects previously attributed to ATM loss, such as defective H2AX-Ser139 phosphorylation and EZH2 expression increase [50, 72], were not observed in our proteomic analysis, perhaps through compensation by other mechanisms [73, 74]. This observation, and the resistance of this tumor to radiotherapy, cautions the current development of ATM pharmacologic inhibitors designed to synergize with radiation-induced DNA damage in cancer cell killing [75]. PARP1 is implicated in DDR and maintenance of genome stability [76] and PARP inhibitors confer synthetic lethality in tumors with *BRCA1/BRCA2* alterations and DNA repair deficiency [77]. F5 was initially treated with PARP inhibitors without response, but did not show recurrent DDR pathway alterations as F10, for whom other therapies were tried. FANCM, a component of the Fanconi anemia complex, was considered a tumor suppressor involved in DDR, until very recent studies showed a novel role in telomere maintenance, specifically in cancer cells with alternative lengthening of telomeres (ALT) [78–80]. Depletion of FANCM in these cells induced reduced cell viability, and hence FANCM inhibition has been proposed as therapeutic strategy specifically in cancer cells with ALT [78–80]. In F12, the *FANCM* p.Q1701* germline mutation, deleting 2 C-terminal domains that may be involved in the ALT phenotype [78], is accompanied by paradoxical mutant allele loss in the tumor, suggesting perhaps a requirement for one functional *FANCM* allele. Although the *FANCM* p.Q1701* mutation is well established as genetic risk factor for breast cancer development [31], there are no studies addressing the *FANCM* somatic events in patient tumors. Therefore, the possibility of distinct roles of FANCM in normal and ALT-exhibiting cancer cells, as well as the significance of the p.Q1701* and other truncating *FANCM* mutations in cancer remain to be elucidated.

The detection of germline mutations in cancer-related genes in all three patients was unexpected, knowing that, despite abundant literature documenting somatic mutations, germline mutations in DMG/K27M have not been similarly explored. A pathogenic germline mutation in *MUTYH*, involved in Lynch syndrome and encoding a DNA base excision repair enzyme, was reported in a pediatric case of spinal cord DMG/K27M [81]. These findings warrant studies on larger cohorts to assess the germline mutation incidence, which might currently be underestimated in DMG/K27M.

The RTK/PI3K/MAPK/mTOR pathway was consistently activated in all cases by proteomic analysis, although only F5 showed *PIK3CA* and additional *TSC1* mutations in the common signature. Focal populations or subpopulations in all three cases acquired a variety of oncogenic genomic alterations during the development of the tumor, including in *PDGFRA*, *KIT, FGFR2*, *KRAS*, *NF1*, *PIK3CA*, *PIK3R1* and *STK11* genes. Except for *PDGFRA* amplification that correlated with PDGFRα overexpression, the proteomic analysis showed more global upregulation or activation of RTKs, including EGFR, PDGFRβ and FGFR2. RTK inhibitors are widely used in the treatment of various cancers, but response requires tumor dependency to the respective RTK signaling, and residual disease develops as a result of tumor cell reprogramming [82]. EGFR inhibitors are successfully used in solid cancers harboring EGFR mutations or overexpression [82], and were used in F12 that showed EGFR overexpression and mild activation. However, mechanisms of rapid EGFR resistance may involve transcriptional upregulation of other RTKs, such as PDGFRβ and FGFR2/3 [83, 84], and we noted high PDGFRβ levels and FGFR2 upregulation without CN gain in F12 foci, explaining therapy resistance. Signaling through RTKs and PI3K/PTEN/PHLPP pathway induces increased cell invasion [85–88], and contributions from upregulated TGFβ/PAI-1 and CD44/c-SRC pathways [54–58] most likely fueled invasion in DIPG. All these pathways are targetable and efforts are underway to test drug inhibitors in glioma cells [28, 54, 89]. However, due to the multitude of activated pathways, and regardless of the epigenetic or therapy-induced mechanism of upregulation of these RTKs, transcription regulators or invasiveness mediators, we believe that more general strategies, either immune or epigenetic, aiming shutdown of aberrant transcriptional programs, such as use of BRD4 inhibitors [90] (Fig. 5c-d), might prove beneficial in DIPG.

## Conclusions

This study contributed several novel genetic alterations, and described a populational heterogeneity not previously appreciated in DMG. The integrated analysis of infratentorial and supratentorial foci allowed outlining migratory pathways and revealed a complex spatiotemporal evolution in DMG that recommends, when applicable, harvesting of both pontine and cerebellar biopsies for accurate populational genetic characterization. The unique proteomic exploration from this study revealed the activation of a multitude of oncogenic and invasion pathways, including positive feedback loops triggered by chemotherapy. In addition to tumor heterogeneity, this global activation that includes cancer cell reprogramming following initial miscellaneous therapeutic attempts explains most likely DMG therapy resistance. This integrated analysis also uncovered previously unknown targetable pathways, strongly supporting the identification of rational combination therapies in DIPG.

## Supplementary information

**Additional file 1 **: **Supplemental Table S1**. Primary antibodies for WB. **Supplemental Table S2**. Autopsy gross parameters. **Supplemental Table S3**. IHC results. **Supplemental Table S4**. Mutations in DIPG. **Supplemental Table S5**. CNVs. **Supplemental Figure S1**. Radiologic characteristics of DIPG cases. A. F5 MRI: axial (left and center) and coronal (right) T2W-FLAIR images showing hyperintense diffusely infiltrating pontine mass extending posteriorly into the right cerebellum and rostrally into the midbrain and subthalamus. Images were acquired 2.5 months pre-mortem. B-C. F10 MRI at initial diagnosis (B) and 10 months later (C) showing rim-enhancing pontocerebellar mass with initial extension into the left cerebellum (B) and subsequent extension into the right cerebellum and frontal periventricular region (C). Yellow arrows show post-contrast enhancing tumor foci. D. F12 axial and sagittal T2W-FLAIR images acquired 1-month pre-mortem showing the frontal and cerebellar secondary foci with yellow arrows. **Supplemental Figure S2**. Example of histologic examination for H&E quantified analysis. F12 H&E sections are shown at the levels schematically illustrated on a normal brain coronal section. Higher magnification of areas marked with small blue squares are shown in adjacent pictures with blue borders. Note massive infiltration of periventricular areas, white matter tracts, septum pellucidum, hippocampus, brainstem and cerebellum (red arrow shows the invasive tumor front in the left cerebellum) by neoplastic cells with pleomorphic nuclei resembling glioblastoma. LV, lateral ventricle; R, right; L, left. **Supplemental Figure S3**. Variable ultrastructural morphology of neoplastic cells in DIPG. A. Variable nuclear morphology of F5 neoplastic cells, showing elongated/fibrillary forms (yellow arrows) admixed with larger forms (blue arrows). The red arrow indicates a myelinated axon “hugged” by a fibrillary neoplastic cell. B. F10 larger neoplastic astrocyte with abundant cytoplasm (blue arrow) dissecting the myelinated axons (red arrow) of the corpus callosum. C. F12 neoplastic astrocytes with nuclear pleomorphism (blue arrows) within the frontal white matter (myelinated axons shown with red arrows and oligodendroglia shown with green arrow). D. F12 large pontine fibrillary neoplastic astrocyte with prominent nucleolus. **Supplemental Figure S4**. Ganglioglioma-like pontine focus in DIPG. F10 H&E of a focal proliferation of large binucleated dysplastic ganglion cells, interspersed with small astrocytic neoplastic cells, conferring a typical ganglioglioma appearance to a limited pontine area of the tumor. IHC and NGS following microdissection of this area revealed histone H3 K27M mutation in all the neoplastic cells, consistent with DMG/K27M. **Supplemental Figure S5**. IHC profiles in DIPG. IHC with indicated antibodies of the pontine tumor areas from F5, F10 biopsy and autopsy, and F12. The F10 autopsy focus of dysplastic binucleated ganglion cells (red arrows) is shown. These cells express GFAP but not vimentin, whereas the small neoplastic cells express both types of intermediate filaments. **Supplemental Figure S6**. Quantitative protein expression analysis in DIPG. A-B. Bar graphs representing the indicated protein levels from various foci of the 3 DIPG autopsies. The quantification of the WB bands was performed by densitometric analysis, as described in Material and Methods. Individual densitometric values were normalized to the corresponding actin values. In (B), the phosphoprotein values were normalized to the corresponding total unphosphorylated protein values, as indicated. Results are expressed as fold-increase or fold-decrease in comparison to normal control. The WBs for each antibody were repeated at least twice, with similar results. The labeling is as in Fig. 4: T, tumor; N, normal; primary pontine (P) foci indicated in red; secondary cerebellar (C) and frontal (Fr) foci, in green. Sample laterality: R, right; L, left. For the tri-methylated histone H3 K27 residue (H3K27Me3) antibody in (A) that showed undetectable expression in both normal and tumor samples, the WB was repeated in the presence of a positive control from an autopsy case of glioblastoma, IDH-mutant, WHO grade IV, known to harbor increased levels of H3 K27 tri-methylation.

## Data Availability

Supporting data for this manuscript are available in the Supplemental Material and upon request to the corresponding author.

## Abbreviations

ALT: Alternative lengthening of telomeres
AT: Ataxia telangiectasia
ATM: Ataxia telangiectasia mutated
C-: Carboxyl
CN: Copy number
CNS: Central nervous system
CSF: Cerebrospinal fluid
CT: Computed tomography
DDR: DNA damage response
GCs: Ganglion-like cells
DIPG: Diffuse intrinsic pontine glioma
DMG: Diffuse midline glioma
EGFR: Epidermal growth factor receptor
ERK/MAPK: Extracellular signal-regulated kinase/mitogen-activated protein kinase
FAK: Focal adhesion kinase
FANCM: Fanconi anemia complementation group M
FFPE: Formalin-fixed paraffin-embedded
FGFR: Fibroblast growth factor receptor
FLAIR: Fluid attenuated inversion recovery
GFAP: Glial fibrillary acidic protein
H3F3A gene: H3.3 histone A/H3 histone family member 3A
H&E: Hematoxylin eosin
IHC: Immunohistochemistry
LOH: Loss of heterozygosity
MRI: Magnetic resonance imaging
N-: Amino
NGS: Next generation sequencing
PAI-1: Plasminogen activator inhibitor 1, encoded by *SERPINE1* gene
PARP: Poly (ADP-Ribose) Polymerase
PDGFR: Platelet-derived growth factor receptor
PI3K p110α/*PIK3CA*: Phosphatidylinositol 3-OH kinase catalytic subunit alpha
PI3K p85/*PIK3R1*: Phosphatidylinositol 3-OH kinase regulatory subunit
PRC: Polycomb repressive complex
RTK: Receptor tyrosine kinase
SNP: Single nucleotide polymorphism
TGF-β: Transforming growth factor β
TMB: Tumor mutation burden
VAF: Variant allele fraction
VUS: Variant of unknown significance
W: Weighted (on MRI sequences T1 and T2)
WB: Western blot
WHO: World Health Organization
