# Supplementary material for: Global activation of oncogenic pathways underlies therapy resistance in diffuse midline glioma
Source: Acta Neuropathol Commun. 2020 Jul 17;8:111. doi: 10.1186/s40478-020-00992-9 (PMC7367358; doi:10.1186/s40478-020-00992-9)
Supplement: Supplementary file 1 — Additional file 1 : Supplemental Table S1. Primary antibodies for WB. Supplemental Table S2. Autopsy gross parameters. Supplemental Table S3. IHC results. Supplemental Table S4. Mutations in DIPG. Supplemental Table S5. CNVs. Supplemental Figure S1. Radiologic characteristics of DIPG cases. A. F5 MRI: axial (left and center) and coronal (right) T2W-FLAIR images showing hyperintense diffusely infiltrating pontine mass extending posteriorly into the right cerebellum and rostrally into the midbrain and subthalamus. Images were acquired 2.5 months pre-mortem. B-C. F10 MRI at initial diagnosis (B) and 10 months later (C) showing rim-enhancing pontocerebellar mass with initial extension into the left cerebellum (B) and subsequent extension into the right cerebellum and frontal periventricular region (C). Yellow arrows show post-contrast enhancing tumor foci. D. F12 axial and sagittal T2W-FLAIR images acquired 1-month pre-mortem showing the frontal and cerebellar secondary foci with yellow arrows. Supplemental Figure S2. Example of histologic examination for H&E quantified analysis. F12 H&E sections are shown at the levels schematically illustrated on a normal brain coronal section. Higher magnification of areas marked with small blue squares are shown in adjacent pictures with blue borders. Note massive infiltration of periventricular areas, white matter tracts, septum pellucidum, hippocampus, brainstem and cerebellum (red arrow shows the invasive tumor front in the left cerebellum) by neoplastic cells with pleomorphic nuclei resembling glioblastoma. LV, lateral ventricle; R, right; L, left. Supplemental Figure S3. Variable ultrastructural morphology of neoplastic cells in DIPG. A. Variable nuclear morphology of F5 neoplastic cells, showing elongated/fibrillary forms (yellow arrows) admixed with larger forms (blue arrows). The red arrow indicates a myelinated axon “hugged” by a fibrillary neoplastic cell. B. F10 larger neoplastic astrocyte with abundant cytoplasm (blue ar [file 40478_2020_992_MOESM1_ESM.pdf]

**Supplemental Table S1**. Primary antibodies for WB.

| **Antibody** | **Cat. No.** | **Source** | **Type** | **MW kDA** |
|---|---|---|---|---|
| β-Actin (AC15) | A5441 | Sigma-Aldrich (St Louis, MO) | Mouse mAb | 42 |
| Akt (pan) (C67E7) | 4691 | Cell Signaling Technology (Danvers, MA) | Rabbit mAb | 60 |
| Phospho-Akt (Ser473) (D9E) | 4060 | Cell Signaling Technology (Danvers, MA) | Rabbit mAb | 60 |
| ASXL1 | ABE400 | MilliporeSigma (Burlington, MA) | Rabbit pAb | 180 |
| CD44 (156-3C11) | 3570 | Cell Signaling Technology (Danvers, MA) | Mouse mAb | 80 |
| Cyclin D1 (92G2) | 2978 | Cell Signaling Technology (Danvers, MA) | Rabbit mAb | 36 |
| EGFR (D38B1) | 4267 | Cell Signaling Technology (Danvers, MA) | Rabbit mAb | 175 |
| Phospho-EGFR (Tyr1068) (D7A5) | 3777 | Cell Signaling Technology (Danvers, MA) | Rabbit mAb | 175 |
| ERK (pan) | E17120/L1 | BD Transduction Laboratories (San Jose, CA) | Mouse mAb | 44/42 |
| Phospho-p44/42 MAPK (Erk1/2) (Thr202/Tyr204) (D13.14.4E) | 4370 | Cell Signaling Technology (Danvers, MA) | Rabbit mAb | 44/42 |
| EZH2 (D2C9) | 5246 | Cell Signaling Technology (Danvers, MA) | Rabbit mAb | 98 |
| FAK | 3285 | Cell Signaling Technology (Danvers, MA) | Rabbit pAb | 125 |
| Phospho-FAK (Tyr397) | 3283 | Cell Signaling Technology (Danvers, MA) | Rabbit mAb | 125 |
| FGFR2 (D4H9) | 11835 | Cell Signaling Technology (Danvers, MA) | Rabbit mAb | 92, 145 |
| Histone H3 (D1H2) | 4499 | Cell Signaling Technology (Danvers, MA) | Rabbit mAb | 17 |
| Histone H3K27Ac (D5E4) | 8173 | Cell Signaling Technology (Danvers, MA) | Rabbit mAb | 17 |
| Tri-Methyl-Histone H3 (Lys27) (C36B11) | 9733 | Cell Signaling Technology (Danvers, MA) | Rabbit mAb | 17 |
| Phospho-Histone H2A.X (Ser139) | 2577 | Cell Signaling Technology (Danvers, MA) | Rabbit pAb | 15 |
| Met (D1C2) | 8198 | Cell Signaling Technology (Danvers, MA) | Rabbit mAb | 145 |
| NF-κB p65 (C22B4) | 4764 | Cell Signaling Technology (Danvers, MA) | Rabbit mAb | 65 |
| NF-κB1 p105/p50 | 3035P | Cell Signaling Technology (Danvers, MA) | Rabbit pAb | 105 & 50 |
| N-Myc (D1V2A) | 84406 | Cell Signaling Technology (Danvers, MA) | Rabbit mAb | 62 |
| p16 INK4A | 10883-1-AP | Proteintech (Chicago, IL) | Rabbit pAb | 16 |
| p27 Kip1 (D69C12) | 3686 | Cell Signaling Technology (Danvers, MA) | Rabbit mAb | 27 |
| p53(DO-7) | 48818 | Cell Signaling Technology (Danvers, MA) | Mouse mAb | 53 |
| PAI-1 (C-9) | sc-5297 | Santa Cruz Biotechnology (Dallas, TX) | Mouse mAb | 48 |
| PARP-1 Antibody (F-2) | sc-8007 | Santa Cruz Biotechnology (Dallas, TX) | Mouse mAb | 116 |
| PDGFRα (D1E1E) | 3174 | Cell Signaling Technology (Danvers, MA) | Rabbit mAb | 190 |
| PDGFRβ (28E1) | 3169 | Cell Signaling Technology (Danvers, MA) | Rabbit mAb | 190 |
| PHLPP1 | A300-660A | Bethyl Laboratories (Montgomery, TX) | Rabbit pAb | 185 |
| PHLPP2 | A300-661A | Bethyl Laboratories (Montgomery, TX) | Rabbit pAb | 185 |
| PTEN (138G6) | 9559 | Cell Signaling Technology (Danvers, MA) | Rabbit mAb | 54 |
| SMAD4 (D3R4N) | 46535 | Cell Signaling Technology (Danvers, MA) | Rabbit mAb | 70 |
| Sox2 (D6D9) | 3579 | Cell Signaling Technology (Danvers, MA) | Rabbit mAb | 35 |
| Src (32G6) | 2123 | Cell Signaling Technology (Danvers, MA) | Rabbit mAb | 60 |
| Phospho-Src Family (Tyr416) (D49G4) | 6943 | Cell Signaling Technology (Danvers, MA) | Rabbit mAb | 60 |
| Stat3 (79D7) | 4904 | Cell Signaling Technology (Danvers, MA) | Rabbit mAb | 79, 86 |
| TCF12/HEB (D-3) | sc-28364 | Santa Cruz Biotechnology (Dallas, TX) | Mouse mAb | 86 |

mAb, monoclonal antibody; pAb, polyclonal antibody

**Supplemental Table S2.** Autopsy gross parameters.

| **Autopsy** | **Time** $^{\text{postmortem}}$ (h) | **Brain weight**<br>(normal for age) | **Tumor foci** | **Herniation** | **Hydrocephalus** |
|---|---|---|---|---|---|
| F5 | 10 | 1100 g<br>(1150 ± 70) | R pons,<br>R cerebellum | R cerebellar<br>tonsil | Asymmetrical: L>R |
| F10 | 11 | 1380 g<br>(1260 ± 40) | L pons,<br>R&L cerebellum,<br>R&L LVs | None | Symmetrical |
| F12 | 24 | 1275 g<br>(1260 ± 40) | R pons,<br>R cerebellum, R LV | None | Symmetrical |

F, female; R, right; L, left; LV, lateral ventricle.

**Supplemental Table S3.** IHC results.

| IHC | F5 | F10 biopsy | F10 autopsy | F12 |
|---|---|---|---|---|
| H3-K27M | + | + | + | + |
| p53 | - | - | - | + |
| IDH1-R132H | - | - | NP | - |
| PTEN | NP | NP | NP | - |
| Olig2 | + | NP | - | NP |
| GFAP | + | + | + | - in most cells |
| Vimentin | - in most cells | NP | + in cells from foci<br>- in ganglion-like and infiltrating cells | - in most cells |

NP, not performed

**Supplemental Table S4.** Mutations in DIPG

| Pathway | Gene | Nucleotide | Amino acid | Effect | Other | Patient |
|---|---|---|---|---|---|---|
| Chromatin remodeling | | | | | | |
| | ASXL1 | c.1934dup | G646fs | Frameshift | | F10 |
| | BCOR | c. 4087_4088delTG | C1363fs | Frameshift | | F12 |
| | H3F3A | c.83A>T | K27M | Missene | | All |
| | KDM6A | c.302_334+41del | H101_A112delinsP | Splice | | F12 |
| DNA damage response | | | | | | |
| | ATM | c.8786+1G>A | | Splice | Germline | F10 |
| | ATM | c.7025G>T | G2342V | Missense | | F10 |
| | ATM | c.8631G>T | L2877F | Missense | | F10 |
| | ATR | c.4352G>C | R1451P | Missense | | F5 |
| | FANCM | c.5101C>T | Q1701* | Stop | Germline | F12 |
| | PPM1D | c.1536del | L513fs | Missense | | F10 |
| | TP53 | c.743G>A | R248Q | Missense | | F5 |
| | TP53 | c.476C>A | A159D | Missense | | F5 |
| | TP53 | c.541C>T | R181C | Missense | | F5 |
| | TP53 | c.960_964dupGAAAC | P322fs | Frameshift | | F5 |
| | TP53 | c.695T>G | I232S | Missense | | F5 |
| | TP53 | c.742C>T | R248W | Missense | | F12 |
| RTK/PIK3CA/MAPK/TSC | | | | | | |
| | PDGFRA | c.114+1G>A | S564L | Missense | | F12 |
| | EGFR | c.1837G>A | A613T | Missense | | F12 |
| | PIK3CA | c.1634A>G | E545G | Missense | | F5 |
| | PIK3CA | c.1624G>A | E542K | Missense | | F10 |
| | PIK3CA | c.1031T>G | V344G | Missense | | F12 |
| | PIK3CA | c.3140A>G | H1047R | Missense | | F12 |
| | PIK3R1 | c.1671_1688del | E558_M563del | In-frame deletion | | F5 |
| | PIK3R1 | | E451_Y452dup | Missense | | F10 |
| | KRAS | c.34G>T | G12C | Missense | | F5 |
| | NF1 | c.4768C>T | R1590W | Missense | | F10 |
| | TSC1 | c.2162delG | R721fs | Frameshift | | F5 |
| Other | | | | | | |
| | MYCN | c.789_797delAGATGA | D264_E266del | In-frame del | Germline | F5 |
| | TCF12 | c. 1055T>G | L352* | Stop gain | | F12 |
| | TCF7L2 | c.746C>A | T249N | Missense | | F5 |
| | CAMTA1 | c. 1833 delCCCCT | T611fs | Frameshift | | F5 |
| | STAT4 | c.580G>T | E194* | Stop gain | | F12 |
| | GRIN2A | c.1652-1G>T | | Splice | | F12 |
| | GALNT12 | c.23G>T | R8L | Missense | | F5 LP |

**Supplemental Table S5.** CNVs.

| Pt | Sample | Chr | Start | Stop | Major CN | Minor CN | Variation | LOH | Band | Genes |
|---|---|---|---|---|---|---|---|---|---|---|
| F5 | Spinal cord | | | | | | | | | |
| | | 1 | 63200 | 931431 | 5 | 2 | gain | 0 | 1p end | |
| | | 1 | 146512830 | 248867391 | 3 | 1 | gain | 0 | 1q | *NTRK1; PIK3C2B; **MDM4**; IKBKE; **H3F3A**; WNT9A/3A; FH; AKT3 |
| | | 2 | 25475070 | 47710051 | 1 | 0 | loss | 1 | 2p23.2-16.3 | *ALK; MSH2 |
| | | 6 | 31543500 | 31797588 | 0 | 0 | loss | 1 | 6p21.33 | TNF |
| | | 6 | 160113480 | 160148381 | 8 | 3 | gain | 0 | | SOD2 3' |
| | | 7 | 101671360 | 101892230 | 1 | 0 | loss | 1 | | **CUX1** |
| | | 7 | 101892328 | 105172741 | 0 | 0 | loss | 1 | | **CUX1** |
| | | 7 | 105172790 | 105207701 | 1 | 0 | loss | 1 | | RINT1 |
| | | 11 | 77784260 | 108168051 | 1 | 0 | loss | 1 | 11q21-22.3 | ***KDM4D**; DYNC2H1 |
| | | 15 | 40462250 | 40462351 | 1 | 0 | loss | 1 | | |
| | | 16 | 48575810 | 89987231 | 1 | 0 | loss | 1 | 16q12-end | *CYLD; **SETD6**; NQO1; **PHLPP2**; ZFHX3; PLCG2; FANCA; MC1R |
| | | 17 | 37855780 | 37884281 | 0 | 0 | loss | 1 | 17q12 | ERBB2 only |
| | | 17 | 74732230 | 74733471 | 7 | 0 | gain | 1 | | SRSF2 only |
| | | 19 | 2226170 | 3094781 | 8 | 4 | gain | 0 | 19p13.3 | **DOT1L** 3'; GNA11 5' |
| | | 19 | 3101010 | 6374881 | 3 | 1 | gain | 0 | 19p13.3 | *GNA11; MAP2K2 |
| | | X | 47006840 | 53448871 | 1 | 0 | loss | 1 | Xp11.3-.22 | *RBM10; GATA1; **KDM5C** |
| | Medulla | | | | | | | | | |
| | | 1 | 154407450 | 248856401 | 4 | 1 | gain | 0 | 1q | *NTRK1; PIK3C2B; **MDM4**; IKBKE; **H3F3A**; WNT9A/3A; FH; AKT3 |
| | | 1 | 248867250 | 248867391 | 9 | 2 | gain | 0 | 1q end | |
| | | 6 | 26028750 | 26205161 | 0 | 0 | loss | 1 | | HIST1H3B; HIST1H1E; HIST1H4E |
| | | 8 | 31497490 | 32585611 | 4 | 2 | gain | 0 | | NRG1 5' |
| | | 9 | 98270430 | 98279091 | 13 | 0 | gain | 1 | | PTCH1 3' |
| | | 10 | 112363970 | 129907545 | 5 | 2 | gain | 0 | 10q25.2-26.13 | TCF7L2; EIF3A; **FGFR2** |
| | | 11 | 94194090 | 108124731 | 1 | 0 | loss | 1 | 11q21-22.3 | ***KDM4D**; DYNC2H1 |
| | | 12 | 92531550 | 92539621 | 0 | 0 | loss | 1 | | **BTG1** |
| | | 15 | 45003730 | 49716781 | 0 | 0 | loss | 1 | | **B2M**; LOC100419583; TRIM69: FGF7 |
| | | 15 | 78841150 | 88671961 | 4 | 2 | gain | 0 | | PSMA4; BCL2A1; ZNF592: NTRK3 5' |
| | | 16 | 48589800 | 89987231 | 1 | 0 | loss | 1 | 16q12-end | *CYLD; **SETD6**; NQO1; **PHLPP2**; ZFHX3; PLCG2; FANCA; MC1R |
| | | 21 | 40181940 | 40194831 | 0 | 0 | loss | 1 | | ETS2 |
| | | 22 | 18212990 | 50710701 | 3 | 1 | gain | 0 | chr 22 gain | |
| | | X | 56295210 | 76708761 | 4 | 2 | gain | 0 | | *AMER1; AR; TAF1 |
| | | X | 153760770 | 153764241 | 0 | 0 | loss | 1 | | G6PD mid gene |
| | R pons | | | | | | | | | |
| | | 1 | 63200 | 931431 | 4 | 2 | gain | 0 | 1p end | |
| | | 1 | 2493100 | 2493251 | 1 | 0 | loss | 1 | | TNFRSF14 mid gene |
| | | 1 | 146512830 | 248867391 | 3 | 1 | gain | 0 | 1q | *NTRK1; PIK3C2B; **MDM4**; IKBKE; **H3F3A**; WNT9A/3A; FH; AKT3 |
| | | 6 | 189070 | 210024 | 3 | 0 | gain | 1 | 6p end | |
| | | 6 | 100053550 | 126078461 | 1 | 0 | loss | 1 | | PRDM1; FOXO3; **HDAC2**; DSE; ROS1 |
| | | 8 | 145743170 | 145813491 | 3 | 1 | gain | 0 | 8q end | ARHGAP39 |
| | | 14 | 22872080 | 22937725 | 4 | 2 | gain | 0 | 14q11.2 | **TRA** |
| | | 16 | 48575810 | 89987231 | 1 | 0 | loss | 1 | 16q12-end | *CYLD; **SETD6;** NQO1; **PHLPP2**; ZFHX3; PLCG2; FANCA; MC1R |
| | | 21 | 15448750 | 30339321 | 3 | 1 | gain | 0 | 21q11-21 | *NRIP1 |
| | | X | 39937080 | 39937181 | 1 | 0 | loss | 1 | | BCOR 3' end |
| | | X | 47006840 | 53449569 | 1 | 0 | loss | 1 | Xp11.3-.22 | *RBM10; GATA1; **KDM5C** |
| | L pons | | | | | | | | | |
| | | 1 | 150607010 | 244773171 | 3 | 1 | gain | 0 | 1q | *NTRK1; PIK3C2B; **MDM4**; IKBKE; **H3F3A**; WNT9A/3A; FH; AKT3 |
| | | 2 | 241664281 | 243664079 | 1 | 0 | loss | 1 | | ING5 mid gene |
| | | 4 | 158987 | 1906125 | 1 | 0 | loss | 1 | 4p | |
| | | 7 | 141645886 | 143635864 | 1 | 0 | loss | 1 | | KEL 5' |
| | | 10 | 134305046 | 136240384 | 1 | 0 | loss | 1 | 10q26.3 end | ***ECHS1** |
| | | 16 | 48575804 | 90983389 | 1 | 0 | loss | 1 | 16q12-end | *CYLD; **SETD6**; NQO1; **PHLPP2**; ZFHX3; PLCG2; FANCA; MC1R |
| | | X | 47545298 | 52929053 | 1 | 0 | loss | 1 | Xp11.3-.22 | *RBM10; GATA1; **KDM5C** |

| | | | | | | | | |
|---|---|---|---|---|---|---|---|---|
| R Midbrain | | | | | | | | |
| 1 | 149900935 | 200055395 | 3 | 1 | gain | 0 | 1q | *NTRK1; PIK3C2B 5' |
| 1 | 203450061 | 205405579 | 4 | 0 | gain | 1 | 1q | PIK3C2B 3' |
| 1 | 208042501 | 245295150 | 3 | 1 | gain | 0 | 1q | ***MDM4**; IKBKE; **H3F3A**; WNT9A/3A; FH; AKT3 |
| 5 | 180652167 | 181642629 | 3 | 1 | gain | 0 | 5q end | TRIM41; RACK1 |
| 6 | 19835940 | 26205204 | 4 | 2 | gain | 0 | | ID4; HIST1H3B; HIST1H1E; HIST1H4E |
| 7 | 13940343 | 19153586 | 3 | 1 | gain | 0 | | ETV1 |
| 9 | 101585518 | 101585727 | 1 | 0 | loss | 1 | | GALNT12 mid segment |
| 10 | 96842061 | 102953600 | 1 | 0 | loss | 1 | 10q | *NKX2-3; FGF8 |
| 10 | 114033376 | 115602874 | 3 | 0 | gain | 1 | 10q26.11 | TCF7L2 |
| 10 | 134305046 | 136240384 | 1 | 0 | loss | 1 | 10q26.3 end | ***ECHS1** |
| 11 | 69633462 | 69633721 | 0 | 0 | loss | 1 | | FGF3 3'end |
| 11 | 94889005 | 102614226 | 1 | 0 | loss | 1 | 11q21-22.3 | ***KDM4D**; DYNC2H1 |
| 14 | 21970547 | 23839257 | 3 | 1 | gain | 0 | 14q11.2 | **TRA** |
| 14 | 106384840 | 106407475 | 1 | 0 | loss | 1 | | IGH mid gene |
| 16 | 48575804 | 90987229 | 1 | 0 | loss | 1 | 16q12-end | *CYLD; **SETD6**; NQO1; **PHLPP2**; ZFHX3; PLCG2; FANCA; MC1R |
| 19 | 30314542 | 30314704 | 0 | 0 | loss | 1 | | CCNE1 3'end |
| 21 | 36164412 | 37510250 | 3 | 0 | gain | 1 | | **RUNX1** |
| 22 | 38367227 | 41489122 | 3 | 0 | gain | 1 | | *SOX10; PDGFB |
| R cerebellum | | | | | | | | |
| 1 | -927228 | 1060009 | 4 | 2 | gain | 0 | 1p end | |
| 1 | 146504886 | 244772895 | 3 | 1 | gain | 0 | 1q incl end | *NTRK1; PIK3C2B; **MDM4**; IKBKE; **H3F3A**; WNT9A/3A; FH; AKT3 |
| 2 | 15095281 | 17072879 | 8 | 0 | gain | 1 | 2p24.3 | MYCNOS; **MYCN** |
| 2 | 127047351 | 129047349 | 1 | 0 | loss | 1 | | |
| 2 | 242800895 | 242801010 | 4 | 0 | gain | 1 | 2q end | PDCD1 3' end |
| 10 | 123237854 | 123357599 | 6 | 0 | gain | 1 | 10q26.13 | **FGFR2 only** |
| 10 | 134292486 | 136278064 | 1 | 0 | loss | 1 | 10q26.3 end | ***ECHS1** |
| 11 | 77790579 | 103339453 | 1 | 0 | loss | 1 | 11q21-22.3 | ***KDM4D**; DYNC2H1 |
| 14 | 21970547 | 23839257 | 4 | 2 | gain | 0 | 14q11.2 | **TRA** |
| 16 | 48573966 | 90987229 | 1 | 0 | loss | 1 | 16q12-end | *CYLD; **SETD6**; NQO1; **PHLPP2**; ZFHX3; PLCG2; FANCA; MC1R |
| L cerebellum | | | | | | | | |
| 1 | 63200 | 91571 | 8 | 3 | gain | 0 | 1p end | |
| 1 | 146504886 | 146505035 | 0 | 0 | loss | 1 | | LOC728989 5' |
| 1 | 248867390 | 248867391 | 4 | 0 | gain | 1 | 1q end | |
| 2 | 242800900 | 242801001 | 7 | 0 | gain | 1 | 2q end | PDCD1 3' end |
| 16 | 48584868 | 89987275 | 1 | 0 | loss | 1 | 16q12-end | *CYLD; **SETD6**; NQO1; **PHLPP2**; ZFHX3; PLCG2; FANCA; MC1R |
| R thalamus | | | | | | | | |
| 1 | 146504886 | 199800076 | 3 | 1 | gain | 0 | | *NTRK1; PIK3C2B 5' |
| 1 | 203440471 | 205434349 | 4 | 0 | gain | 1 | | PIK3C2B 3' |
| 1 | 208044447 | 248856440 | 3 | 1 | gain | 0 | | ***MDM4**; IKBKE; **H3F3A**; WNT9A/3A; FH; AKT3 |
| 2 | 29416070 | 30143545 | 1 | 0 | loss | 1 | 2p23.2-23.1 | ALK only |
| 6 | 31543499 | 31670409 | 0 | 0 | loss | 1 | 6p21.33-21.32 | *TNF; TAP2/1; **DAXX** |
| 16 | 48573966 | 89987275 | 1 | 0 | loss | 1 | 16q12-end | *CYLD; **SETD6**; NQO1; **PHLPP2**; ZFHX3; PLCG2; FANCA;MC1R |
| 17 | 37855777 | 38504736 | 1 | 0 | loss | 1 | 17q12 | *ERBB2 |
| 21 | 15448744 | 30339365 | 3 | 1 | gain | 0 | 21q11-21 | *NRIP1 |
| X | 47522255 | 52934116 | 1 | 0 | loss | 1 | Xp11.3-.22 | *RBM10; GATA1; **KDM5C** |
| Splenium CC | | | | | | | | |
| 8 | 145813390 | 145813491 | 5 | 2 | gain | 0 | 8q end | ARHGAP39 3'end |
| 11 | 102987280 | 103836791 | 1 | 0 | loss | 1 | 11q22.3 | DYNC2H1 only |
| 17 | 700930 | 8141891 | 3 | 1 | gain | 0 | 17p13.3-13.1 | ***CRK**; FGF11; **TP53**; AURKB; CTC1 |
| 17 | 66547250 | 80840621 | 3 | 1 | gain | 0 | 17q25 | *SOX9; NHERF1; GRB2; SRSF2; RPTOR |
| 19 | 426900 | 8468321 | 3 | 1 | gain | 0 | 19p | *SHC2; FGF22; TCF3; **DOT1L**; GNA11; MAP2K2; CD70 |
| 19 | 35837040 | 36214181 | 0 | 0 | loss | 1 | | *COX6B1; **KMT2B** 5' |
| X | 296370 | 1315021 | 4 | 2 | gain | 0 | Xp end | PPP2R3B |
| X | 44970600 | 53432841 | 1 | 0 | loss | 1 | Xp11.3-.22 | *RBM10; GATA1; **KDM5C** |

F10 Pons ganglion cell-like focus

| | | | | | | | | |
|---|---|---|---|---|---|---|---|---|
| 1 | 146512830 | 248867391 | 3 | 1 | gain | 0 | 1q | *NTRK1; PIK3C2B; **MDM4**; IKBKE; **H3F3A**; WNT9A/3A; FH; AKT3 |
| 1 | 248867390 | 248867391 | 4 | 0 | gain | 1 | 1q end | |
| 3 | 164905600 | 197203701 | 4 | 2 | gain | 0 | 3q | *PIK3CA; **SOX2**; BCL6; TP63; HES1 |
| 3 | 197203750 | 197203751 | 3 | 0 | gain | 1 | 3q end | |
| 4 | 54882630 | 55606981 | 4 | 1 | gain | 0 | 4q12 | **PDGFRA; KIT** |
| 6 | 43746600 | 44232141 | 4 | 0 | gain | 1 | | VEGFA 3'; SLC35B2; NFKBIE |
| 8 | 145813490 | 145813491 | 3 | 0 | gain | 1 | 8q end | ARHGAP39 3'end |
| 10 | 131565040 | 135281671 | 1 | 0 | loss | 1 | 10q26.3 end | ***ECHS1** |
| 14 | 20834740 | 106580691 | 1 | 0 | loss | 1 | chr 14 loss | |
| 15 | 40498370 | 40500971 | 1 | 0 | loss | 1 | | BUB1B mid segm |
| 15 | 41001210 | 41022191 | 1 | 0 | loss | 1 | | RAD51 3' |
| 17 | 700930 | 7569531 | 3 | 1 | gain | 0 | 17p13.3-13.1 | ***CRK**; FGF11 (no TP53) |
| 17 | 38146080 | 38512481 | 3 | 1 | gain | 0 | | PSMD3; RARA |
| 17 | 62008730 | 80840621 | 3 | 1 | gain | 0 | 17q25 | *GNA13; AXIN2; PRKAR1A; SOX9; NHERF1; GRB2; SRSF2; RPTOR |

Biopsy Pons

| | | | | | | | | |
|---|---|---|---|---|---|---|---|---|
| 1 | | | 3 | 1 | gain | 0 | 1q | *NTRK1; PIK3C2B; **MDM4**; IKBKE; **H3F3A**; WNT9A/3A; FH; AKT3 |
| 11 | | | 2 | 0 | neutral | 1 | | ***ATM** |
| 14 | | | 1 | 0 | loss | 1 | chr 14 loss | |
| 17 | | | 3 | 1 | gain | 0 | 17q25 | *GNA13; AXIN2; PRKAR1A; SOX9; NHERF1; GRB2; SRSF2; RPTOR |

Biopsy Corpus Callosum (anterior)

| | | | | | | | | |
|---|---|---|---|---|---|---|---|---|
| 1 | | | 3 | 1 | gain | 0 | 1q | *NTRK1; PIK3C2B; **MDM4**; IKBKE; **H3F3A**; WNT9A/3A; FH; AKT3 |
| 3 | | | | | gain | | 3q | *PIK3CA; **SOX2**; BCL6; TP63; HES1 |
| 7 | | | | | gain | 0 | 7q | |
| 10 | | | 1 | 0 | loss | 1 | 10q26.3 end | ***ECHS1** |
| 12 | | | 1 | 0 | loss | 1 | 12p | |
| 15 | | | 1 | 0 | loss | 1 | 15q proximal | |
| 20 | | | 1 | 0 | loss | 1 | 20p | |

F12 Pons

| | | | | | | | | |
|---|---|---|---|---|---|---|---|---|
| 1 | 63200 | 63736 | 9 | 3 | gain | 0 | 1p end | |
| 2 | 47596200 | 47596931 | 1 | 0 | loss | 1 | | EPCAM 5' |
| 3 | 37034730 | 37035901 | 4 | 2 | gain | 0 | | EPM2AIP1 |
| 7 | 38296700 | 38298321 | 5 | 2 | gain | 0 | p14.1 | TRG 5' |
| 7 | 66460670 | 107434961 | 3 | 1 | gain | 0 | 7q21.11 to: | *HGF; **CDK6**; **SERPINE1**; **CUX1**; RINT1; PIK3CG; SLC26A3 |
| 7 | 116335790 | 158827481 | 1 | 0 | loss | 1 | 7q31.2 to end | *MET; CFTR; POT1; SMO; BRAF; KEL **EZH2; KMT2C**; SHH |
| 8 | 32463060 | 32583044 | 4 | 2 | gain | 0 | | NRG1 mid segm |
| 10 | 115808330 | 123357211 | 3 | 1 | gain | 0 | 10q26.11 | EIF3A only |
| 11 | 118307210 | 118307661 | 0 | 0 | loss | 1 | | **KMT2A** 5' |
| 12 | 124824740 | 124827791 | 3 | 1 | gain | 0 | | NCOR2 mid segm gain |
| 12 | 124829130 | 124831391 | 0 | 0 | loss | 1 | | NCOR2 mid segm loss; contiguous |
| 14 | 35873610 | 106580691 | 1 | 0 | loss | 1 | 14q | *NKX2-1; PAX9; **FANCM**; HIF1A; MAX; RAD51B; MLH3; DICER1 |
| 16 | 31107720 | 89987231 | 1 | 0 | loss | 1 | 16q12-end | *CYLD; **SETD6**; NQO1; **PHLPP2**; ZFHX3; PLCG2; FANCA; MC1R |
| 17 | 700930 | 8151341 | 2 | 0 | neutral | 1 | 17p13.3-13.1 | ***CRK**; FGF11; **TP53**; AURKB; CTC1 |
| 18 | 193090 | 77213191 | 1 | 0 | loss | 1 | chr 18 loss | ***SMAD4**; **PHLPP1** |
| 19 | 1219310 | 1227871 | 0 | 0 | loss | 1 | | **STK11** |
| 20 | 351870 | 25276331 | 1 | 0 | loss | 1 | 20p | *PAX1 |
| 21 | 36259120 | 37507781 | 0 | 0 | loss | 1 | | **RUNX1 3'** |

L cerebellum

| | | | | | | | | |
|---|---|---|---|---|---|---|---|---|
| 1 | 63200 | 931431 | 4 | 2 | gain | 0 | 1p end | |
| 4 | 55124910 | 55561911 | 15 | 0 | gain | 1 | 4q12 | **PDGFRA** |
| 4 | 55561960 | 55606981 | 12 | 0 | gain | 1 | 4q12 | **KIT** |
| 6 | 189070 | 210024 | 3 | 1 | gain | 0 | 6p end | |
| 6 | 137527460 | 144274241 | 1 | 0 | loss | 1 | | IFNGR1; TNF!IP3; ECT2L; PLAGL1 |
| 7 | 6018310 | 28106961 | 3 | 1 | gain | 0 | | *PMS2; RAC1; ETV1: JAZF1 contiguos with: |
| 7 | 30673470 | 38325601 | 4 | 2 | gain | 0 | p14.1 | GARS; TRG |

| | | | | | | | | |
|---|---|---|---|---|---|---|---|---|
| 7 | 80374190 | 107434961 | 4 | 1-0 | gain | 0-1 | 7q21.11 to: | *HGF; **CDK6**; **SERPINE1**; **CUX1**; RINT1; PIK3CG; SLC26A3 |
| 7 | 116335790 | 158827481 | 1 | 0 | loss | 1 | 7q31.2 to end | *MET; CFTR; POT1; SMO; BRAF; KEL **EZH2; KMT2C**; SHH |
| 9 | 211400 | 137313611 | 3 | 1 | gain | 0 | chr 9 gain (incl CDKN2A/B) except 9q end | |
| 10 | 268340 | 135288881 | 1 | 0 | loss | 1 | chr 10 loss | |
| 14 | 22872080 | 22937691 | 4 | 2 | gain | 0 | 14q11.2 | TRA |
| 14 | 45605220 | 106580691 | 1 | 0 | loss | 1 | 14q | ***FANCM**; HIF1A; MAX; RAD51B; MLH3; DICER1 |
| 16 | 48589800 | 89987231 | 1 | 0 | loss | 1 | 16q12-end | *CYLD; **SETD6**; NQO1; **PHLPP2**; ZFHX3; PLCG2; FANCA; MC1R |
| 17 | 700930 | 1356603 | 2 | 0 | neutral | 1 | 17p13.1 | ***CRK** |
| 17 | 1356610 | 19619881 | 1 | 0 | loss | 1 | 17p13.1-11.2 | *FGF11; **TP53**; AURKB; CTC1; MAP2K4; NCOR1; FLCN |
| 17 | 29422400 | 33430561 | 3 | 1 | gain | 0 | | NF1; **SUZ12**; RAD51 5' |
| 17 | 80840520 | 80840621 | 3 | 0 | gain | 1 | 17q end | TBCD 3' last segm |
| 18 | 193090 | 320061 | 3 | 0 | gain | 1 | | USP14; THOC1; COLEC12 contiguous w |
| 18 | 649830 | 673341 | 5 | 2 | gain | 0 | 18p end (p11.32) | C180rf56; TYMSOS; **TYMS** |
| 18 | 13578550 | 77213191 | 1 | 0 | loss | 1 | chr18 loss | ***SMAD4**; **PHLPP1** |
| 19 | 58596860 | 59067621 | 1 | 0 | loss | 1 | 19q end | |
| 20 | 351870 | 25276344 | 1 | 0 | loss | 1 | 20p | *PAX1 |
| 21 | 15454530 | 30339271 | 3 | 1 | gain | 0 | 21q11-21 | *NRIP1 |
| R frontal | | | | | | | | |
| 4 | 55127250 | 55594261 | 4 | 0 | gain | 1 | 4q12 | **PDGFRA** |
| 4 | 55594300 | 55606981 | 4 | 0 | gain | 1 | 4q12 | **KIT** |
| 6 | 170242880 | 170242981 | 4 | 0 | gain | 1 | 6q end | |
| 7 | 38296700 | 38298321 | 5 | 2 | gain | 0 | p14.1 | TRG 5' |
| 7 | 80374190 | 107434961 | 4 | 1-0 | gain | 0-1 | 7q21.11 to: | *HGF; **CDK6**; **SERPINE1**; **CUX1**; RINT1; PIK3CG; SLC26A3 |
| 7 | 116335790 | 158827481 | 1 | 0 | loss | 1 | 7q31.2 to end | *MET; CFTR; POT1; SMO; BRAF; KEL **EZH2; KMT2C**; SHH |
| 14 | 22872080 | 22909291 | 4 | 2 | gain | 0 | 14q11.2 | **TRA** |
| 14 | 45605220 | 106580691 | 1 | 0 | loss | 1 | 14q | ***FANCM**; HIF1A; MAX; RAD51B; MLH3; DICER1 |
| 16 | 48573970 | 89987231 | 1 | 0 | loss | 1 | 16q12-end | *CYLD; **SETD6**; NQO1; **PHLPP2;** ZFHX3; PLCG2; FANCA; MC1R |
| 17 | 700930 | 19619881 | 1 | 0 | loss | 1 | 17p13.1-11.2 | ***CRK**; FGF11; **TP53**; AURKB; CTC1; MAP2K4; NCOR1; FLCN |
| 17 | 80840520 | 80840621 | 4 | 0 | gain | 1 | 17q end | TBCD 3' last segm |
| 18 | 649830 | 673341 | 5 | 2 | gain | 0 | 18p end (p11.32) | C180rf56; TYMSOS; **TYMS** |
| 18 | 13578550 | 77213191 | 1 | 0 | loss | 1 | chr18 loss | ***SMAD4**; **PHLPP1** |
| 19 | 58596860 | 59067621 | 1 | 0 | loss | 1 | 19q end | |
| 20 | 351870 | 378351 | 3 | 1 | gain | 0 | 20p end | |
| 20 | 1115850 | 25276344 | 1 | 0 | loss | 1 | 20p | *PAX1 |
| X | 129154940 | 129168661 | 4 | 0 | gain | 1 | | BCORL1 mid gene |

Recurrent CNVs yellow
F5-only recurrent CNVs, in shades of green
F10-only recurrent CNV, in blue
F12-only recurrent CNVs, in shades of purple
10q focal gain, in bright green
14 and 14q loss, in light orange
17p CNVs, in dark orange
* multiple genes, some of which are mentioned
Genes discussed in this report, in bold
Start and stop nucleotide positions are given relative to GRCh37/hg19

# SUPPLEMENTAL FIGURES

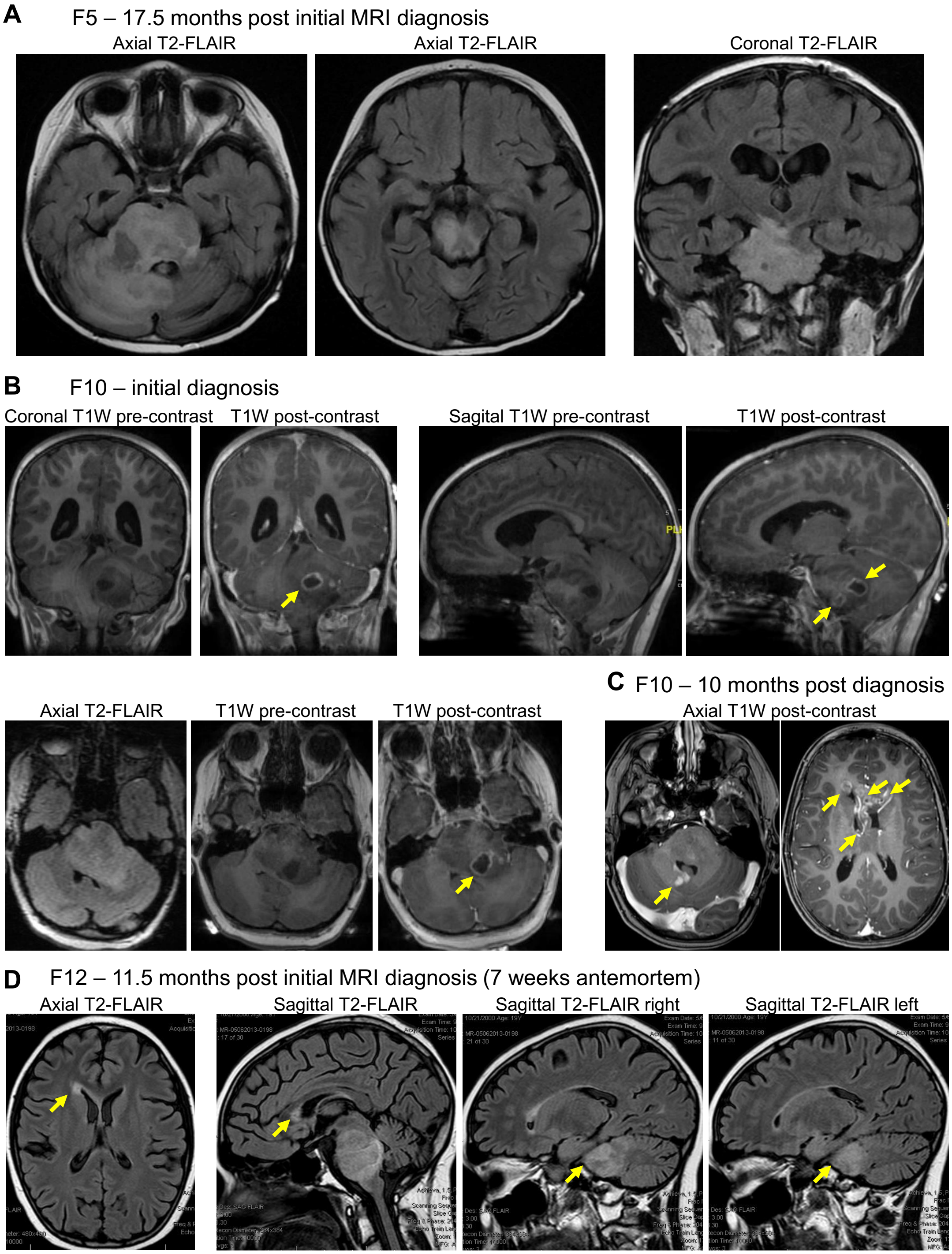


**Supplemental Figure S1.** Radiologic characteristics of DIPG cases. **A.** F5 MRI: axial (left and center) and coronal (right) T2W-FLAIR images showing hyperintense diffusely infiltrating pontine mass extending posteriorly into the right cerebellum and rostrally into the midbrain and subthalamus. Images were acquired 2.5 months pre-mortem. **B-C.** F10 MRI at initial diagnosis (B) and 10 months later (C) showing rim-enhancing pontocerebellar mass with initial extension into the left cerebellum (B) and subsequent extension into the right cerebellum and frontal periventricular region (C). Yellow arrows show post-contrast enhancing tumor foci. **D.** Axial and sagittal T2W-FLAIR images acquired 1-month pre-mortem showing the frontal and cerebellar secondary foci with yellow arrows.

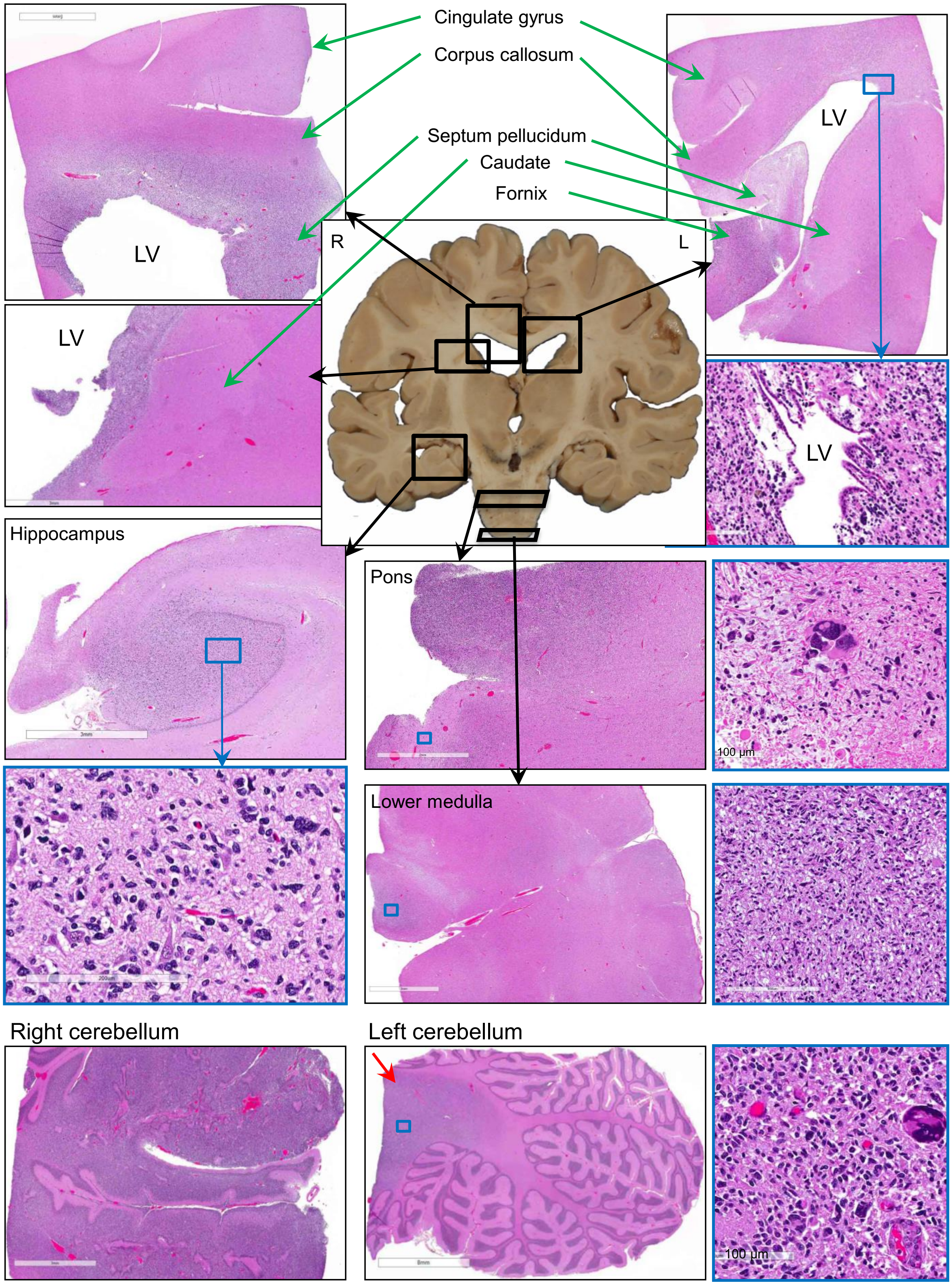


**Supplemental Figure S2.** Example of histologic examination for H&E quantified analysis. F12 H&E sections are shown at the levels schematically illustrated on a normal brain coronal section. Higher magnification of areas marked with small blue squares are shown in adjacent pictures with blue borders. Note massive infiltration of periventricular areas, white matter tracts, septum pellucidum, hippocampus, brainstem and cerebellum (red arrow shows the invasive tumor front in the left cerebellum) by neoplastic cells with pleomorphic nuclei resembling glioblastoma. LV, lateral ventricle; R, right; L, left.

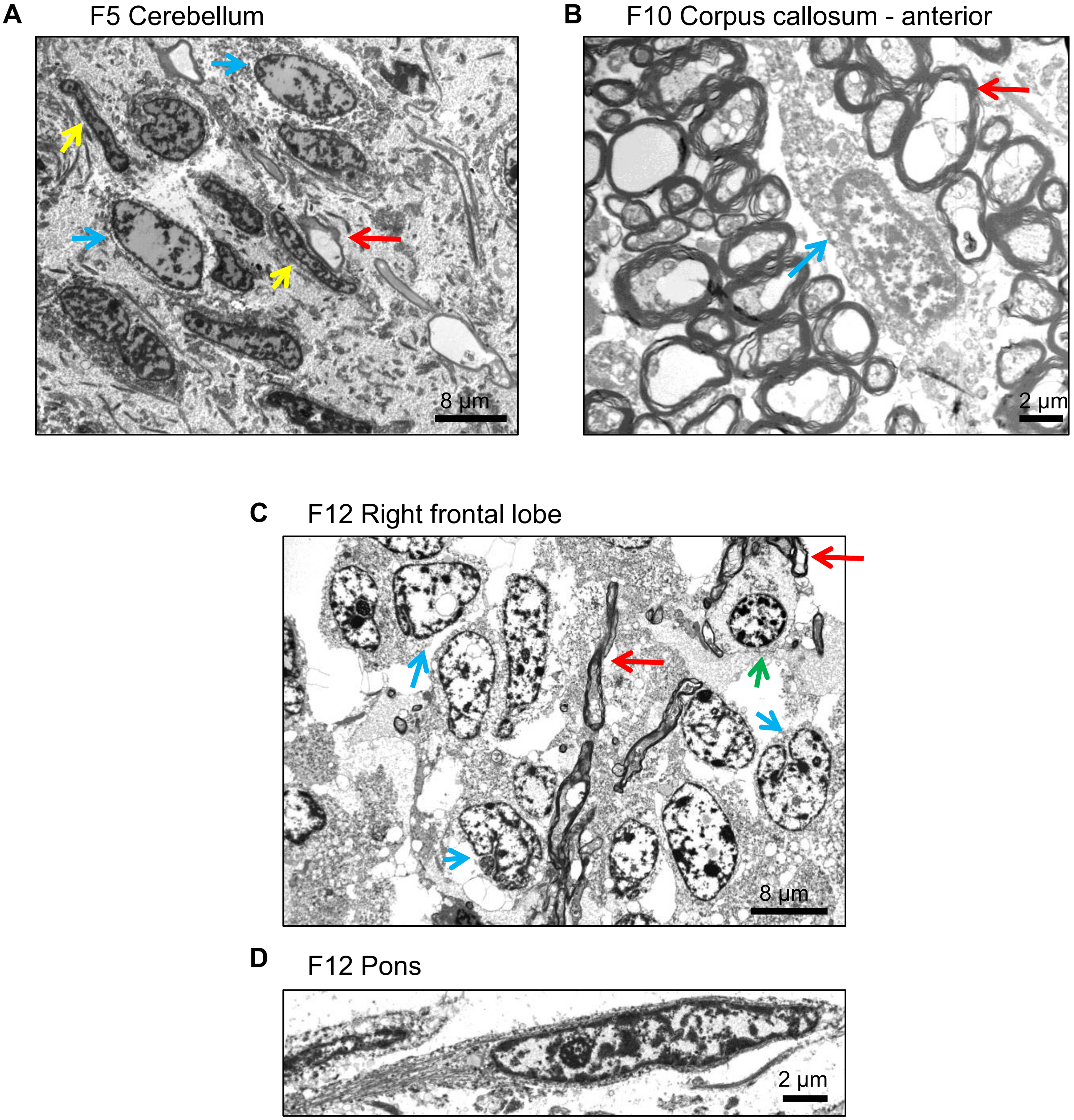


**Supplemental Figure S3.** Variable ultrastructural morphology of neoplastic cells in DIPG. **A.** Variable nuclear morphology of F5 neoplastic cells, showing elongated/fibrillary forms (yellow arrows) admixed with larger forms (blue arrows). The red arrow indicates a myelinated axon “hugged” by a fibrillary neoplastic cell. **B.** F10 larger neoplastic astrocyte with abundant cytoplasm (blue arrow) dissecting the myelinated axons (red arrow) of the corpus callosum. **C.** F12 neoplastic astrocytes with nuclear polymorphism (blue arrows) within the frontal white matter (myelinated axons shown with red arrows and oligodendroglia shown with green arrow). **D.** F12 large pontine fibrillary neoplastic astrocyte with prominent nucleolus.

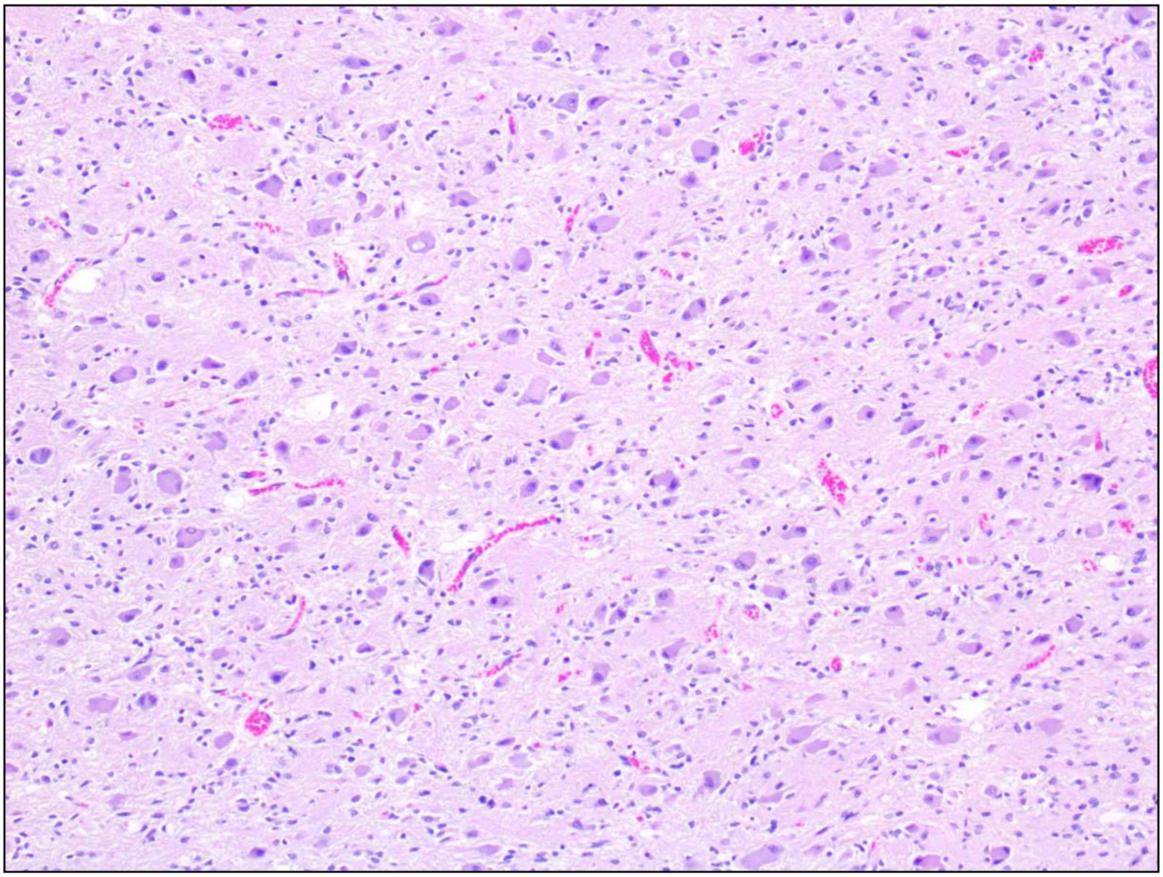

**Supplemental Figure S4.** Ganglioglioma-like pontine focus in DIPG. F10 H&E of a focal proliferation of large binucleated dysplastic ganglion cells, interspersed with small astrocytic neoplastic cells, conferring a typical ganglioglioma appearance to a limited pontine area of the tumor. IHC and NGS following microdissection of this area revealed histone H3 K27M mutation in all the neoplastic cells, consistent with DMG/K27M.

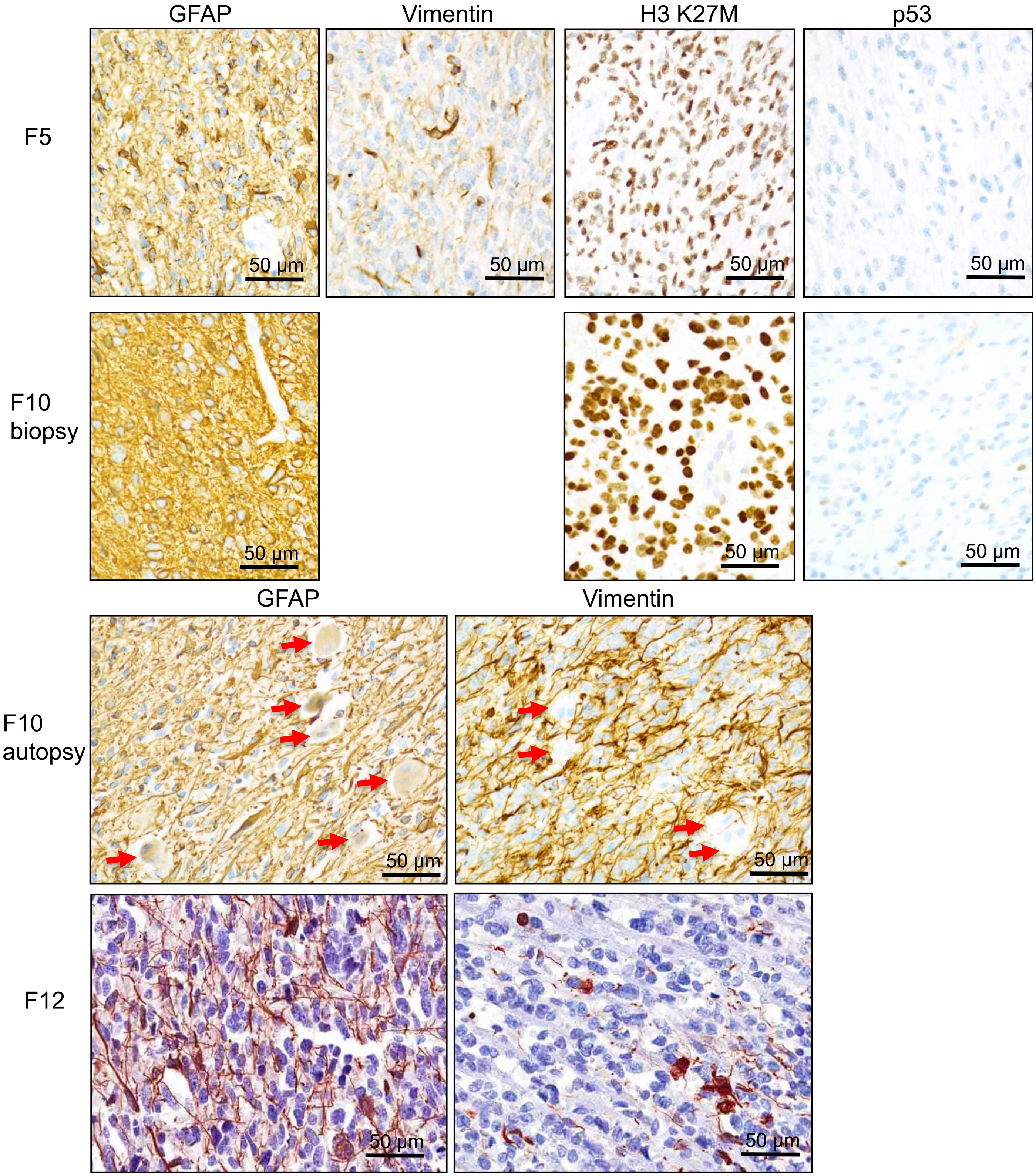


**Supplemental Figure S5.** IHC profiles in DIPG. IHC with indicated antibodies of the pontine tumor areas from F5, F10 biopsy and autopsy, and F12. The F10 autopsy focus of dysplastic binucleated ganglion cells (red arrows) is shown. These cells express GFAP but not vimentin, whereas the small neoplastic cells express both types of intermediate filaments.

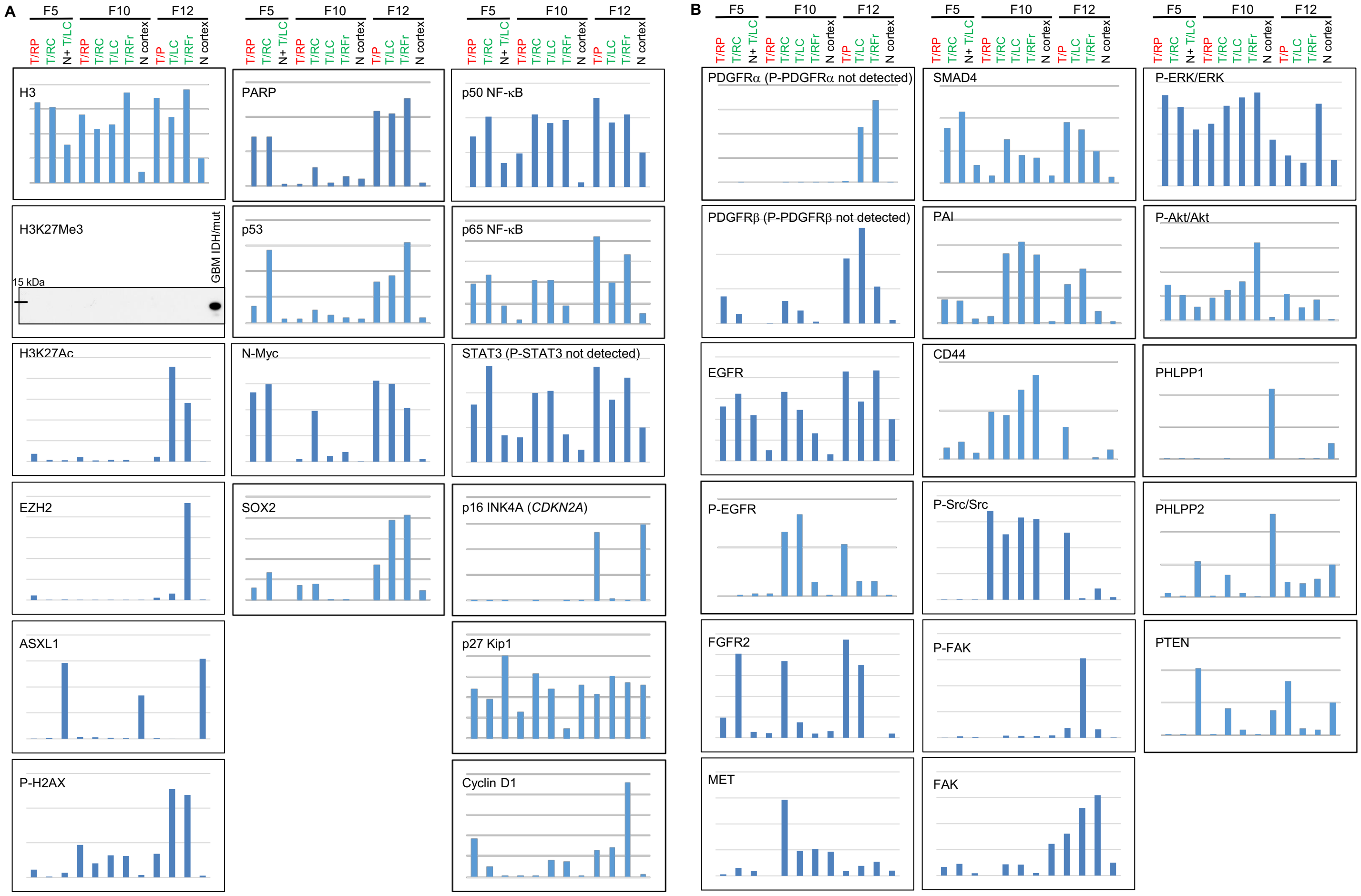


**Supplemental Figure S6.** Quantitative protein expression analysis in DIPG. **A-B**. Bar graphs representing the indicated protein levels from various foci of the 3 DIPG autopsies. The quantification of the WB bands was performed by densitometric analysis, as described in Materia and Methods. Individual densitometric values were normalized to the corresponding actin values. In (B), the phosphoprotein values were normalized to the corresponding total unphosphorylated protein values, as indicated. Results are expressed as fold-increase or fold-decrease in comparison to normal control. The WBs for each antibody were repeated at least twice, with similar results. The labeling is as in Figure 4: T, tumor; N, normal; primary pontine (P) foci indicated in red; secondary cerebellar (C) and frontal (Fr) foci, in green. Sample laterality: R, right; L, left. For the tri-methylated histone H3 K27 residue (H3K27Me3) antibody in (A) that showed undetectable expression in both normal and tumor samples, the WB was repeated in the presence of a positive control from an autopsy case of glioblastoma, IDH-mutant, WHO grade IV, known to harbor increased levels of H3 K27 tri-methylation.
